# Empirical investigation of friction weakening of terrestrial and Martian landslides using discrete element models

**DOI:** 10.1007/s10346-019-01140-8

**Published:** 2019-03-01

**Authors:** Timur Borykov, Daniel Mège, Anne Mangeney, Patrick Richard, Joanna Gurgurewicz, Antoine Lucas

**Affiliations:** 1 Institute of Geological Sciences, Polish Academy of Sciences, Research Centre in Wrocław, Wrocław, Poland; 2 Space Research Centre, Polish Academy of Sciences, Warsaw, Poland; 3 Institut de Physique du Globe de Paris, Sorbonne Paris Cité, Université Paris Diderot, CNRS UMR 7154, Paris, France; 4 ANGE team, INRIA, Lab. J. Louis Lions, Paris, France; 5 IFSTTAR, MAST/GPEM, 44340 Bouguenais, France

**Keywords:** Landslide, Friction, Granular media, Discrete-element modelling, Dam-break flows, Mars, Valles Marineris

## Abstract

Understanding what controls the travelling distance of large landslides has been the topic of considerable debate. By combining observation and experimental data with depth-averaged continuum modelling of landslides and generated seismic waves, it was empirically observed that lower effective friction had to be taken into account in the models to reproduce the dynamics and runout distance of larger volume landslides. Moreover, such simulation and observation results are compatible with a friction weakening with velocity as observed in earthquake mechanics. We investigate here as to whether similar empirical reduced friction should be put into discrete element models (DEM) to reproduce observed runout of large landslides on Earth and on Mars. First we show that, in the investigated parameter range and for a given volume, the runout distance simulated by 3D DEM is not much affected by the number (i.e. size) of grains once this number attains ~ 8000. We then calibrate the model on laboratory experiments and simulate other experiments of granular flows on inclined planes, making it possible for the first time to reproduce the observed effect of initial volume and aspect ratio on runout distances. In particular, the normalised runout distance starts to depend on the volume involved only above a critical slope angle > 16–19°, as observed experimentally. Finally, based on field data (volume, topography, deposit), we simulate a series of landslides on simplified inclined topography. The empirical friction coefficient, calibrated to reproduce the observed runout for each landslide, is shown to decrease with increasing landslide volume (or velocity), going down to values as low as 0.1–0.2. No distinguishable difference is observed between the behaviour of terrestrial and Martian landslides.

**Electronic supplementary material:**

The online version of this article (10.1007/s10346-019-01140-8) contains supplementary material, which is available to authorized users.

## Introduction

**Fig. 1:**
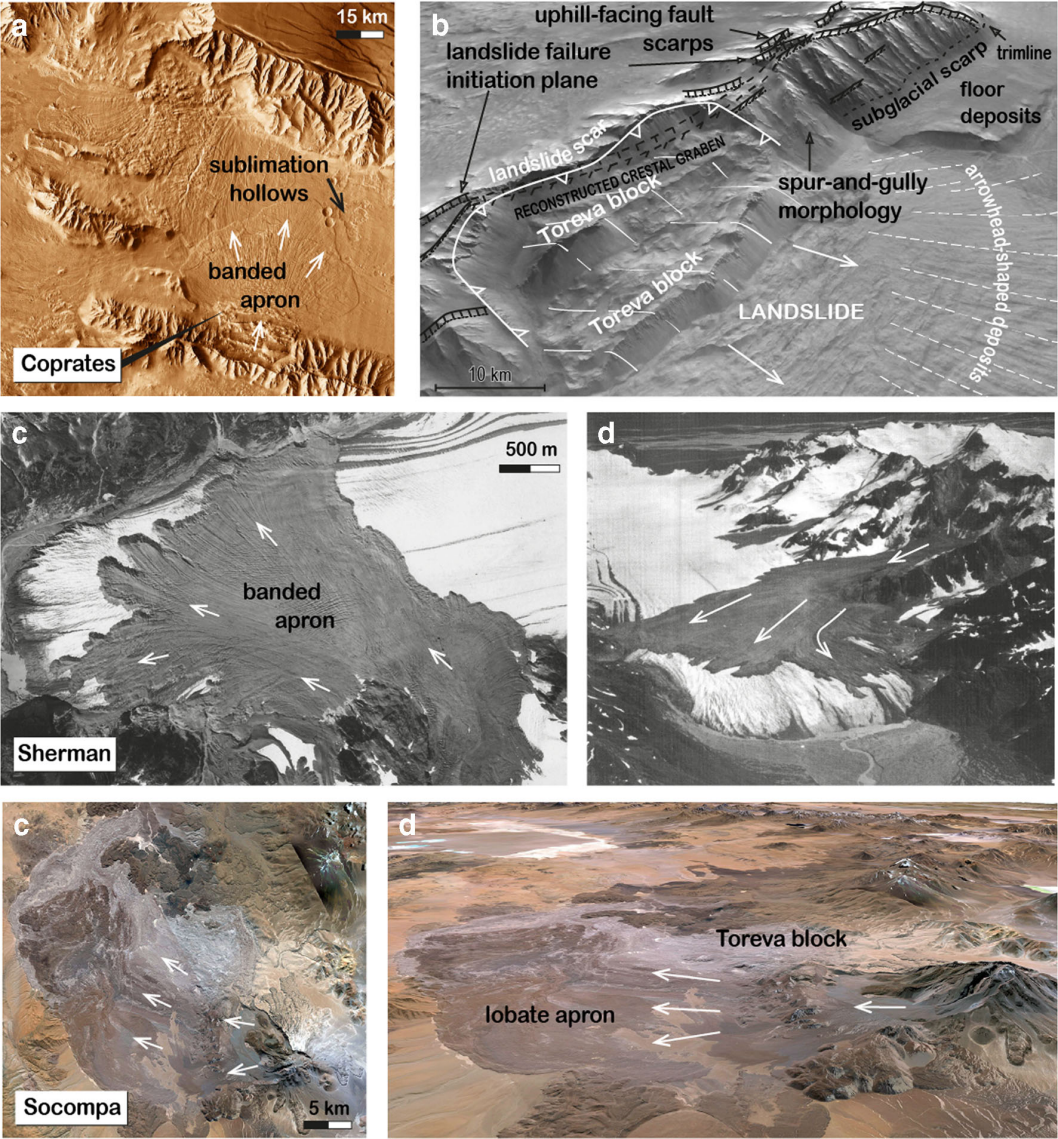
Landslides in Coprates Chasma, in Valles Marineris, Mars (**a**, **b**), compared with the Sherman (**c**, **d**) and Socompa (**e**, **f**) landslides. The Socompa and Sherman landslides, as well as the Coprates Chasma landslide displayed at the bottom of **a** and on **b**, and others, are investigated in this paper (Figs. 13, 14 and 15). Photographs of the Sherman landslide are, on the right and on the left by McSaveney (in Hewitt et al. 2014) and Post (1967), respectively. The Coprates Chasma image mosaic is made from THEMIS images (NASA/JPL/ASU), and interpretation is after Mège and Bourgeois (2011); the Socompa satellite views are from Digital Globe on Google Earth

Rock and debris avalanches of large volumes can travel very long distances along almost flat topographies and represent a high risk for populations (Legros 2002). Numerical modelling of these complex granular flows helps in understanding and predicting such events. Mathematical and numerical models are however based on very simplified description of the real processes involved. Indeed, simulating real landslides on complex topography requires high computational cost, but also, and more critical, the physical processes involved are largely unknown and the hypotheses proposed for explaining the high mobility of real landslides on Earth are hardly quantified (e.g. Legros 2002; Lucas et al. 2014; Delannay et al. 2017). These hypotheses include bulk fluidisation or lubrication by air, gas, water, ice, heating or acoustic waves (see, e.g. references in Shreve 1987; Goren et al. 2010; Ferri et al. 2011; Bulmer 2012; Liu et al. 2015; Mitchell et al. 2015; Charrière et al. 2016; Johnson et al. 2016; Wang et al. 2018), or the presence of an erodible bed (e.g. Mangeney et al. 2010; Crosta et al. 2009, 2015; Iverson et al. 2011; Farin et al. 2014). The behaviour of natural large landslides, which display a wide range of geomorphologic diversity and emplacement conditions (Fig. 1), possibly results from a combination of these processes. Up to now, no consensus exists on the relative impact of these processes on landslide dynamics and deposit because of the lack their quantification.

The identification of the physical processes at work during landslide emplacement is of particular importance on Mars where the presence of water is a key issue. As an example, the high mobility of Martian landslides led Lucchitta (1981, 1987) to advocate wet debris flows. McEwen (1989) argued in favour of dry rock avalanches instead, and Lucchitta et al. (1992) suggested that they may have initiated as dry flows that incorporated water and ended up as mud flows. More generally, as for terrestrial landslides, several hypotheses have been proposed to explain the high mobility of Martian landslide such as fluidisation by liquid or gas in Valles Marineris (Harrison and Grimm 2003; Johnson and Campbell 2017), lubricating due to the presence of clays (Watkins et al. 2015) or ice (Lucchitta 1979, 1981; De Blasio 2011; Mazzanti et al. 2016; De Blasio and Crosta 2017; Crosta et al. 2018), the impact of seismic triggering with activation or reactivation of summital faults (Mège and Bourgeois 2011). Johnson and Campbell (2017) suggest on the contrary that Martian landslides may be less mobile than landslides on Earth, while Lucas et al. (2014) found no clear difference between empirical friction weakening with volume (or velocity) for terrestrial and Martian landslides. Indeed, even huge Martian landslides with scars up to 100 km in length in Ius Chasma, much larger than the broadest scars on Earth (e.g. several kilometres for Socompa landslide), follow the same trend as all other landslides on Earth.

**Fig. 2:**
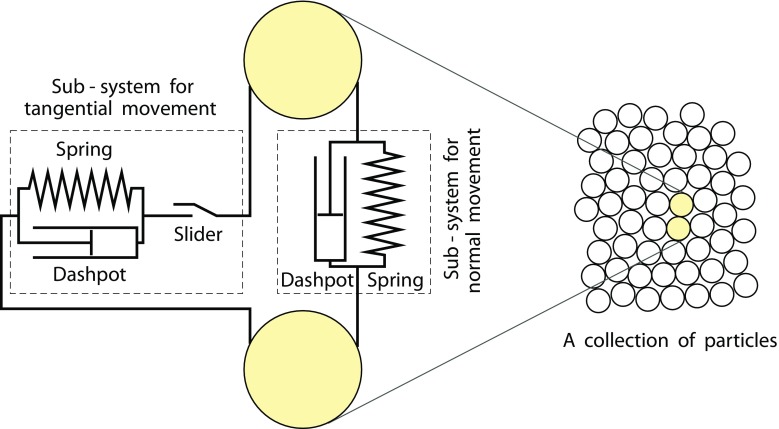
The contact model between two spheres: linear spring–dashpot system

The most usual way to quantify the effective or mean friction, i.e. the inverse of the mobility, is to calculate the Heim’s ratio *H*/*L* between the drop height *H* and the runout distance *L*. However, this ratio is different from the effective friction even though its overall behaviour looks similar (see Equations (1) and (2) and Figures 2b, 2c of Lucas et al. 2014). A more advanced empirical approach is to (i) use a granular flow model that takes topography and deformation of the mass along the slope into account and (ii) calibrate the friction coefficient in the model to reproduce the observed deposit and dynamics of landslides. This approach makes it possible to investigate empirically how the effective friction has to change as a function of the landslide characteristics such as volume, velocity and strain rate in order to reproduce the observed runout distance.

By combining observation and experimental data with depth-averaged continuum modelling of landslides, Lucas et al. (2014) observed that lower effective friction coefficient (i.e. the tangent of the effective friction angle) had to be taken into account in the models when trying to reproduce the runout distance of larger volume landslides. The decrease of effective friction as a function of the volume involved followed on average a common trend for all the landslides they considered on Earth but also on other planets (their Figure 2). By relating this effective friction with the flow velocity calculated by the model, they suggest that these observations are compatible with a friction weakening with velocity (and/or with other related variables such as strain rate) and displacement as observed in earthquake mechanics and laboratory experiments (Di Toro et al. 2011; Rubino et al. 2017). Comparison between seismic data and numerical modelling based on the depth-averaged models suggests a similar decrease of the friction coefficient as a function of the volume involved (Levy et al. 2015). In particular, such small friction coefficients are recovered when trying to match the simulated force history that the landslide applies to the ground to the force history inverted from seismic data. However, these depth-averaged continuum models are generally based on strong approximations such as prescribed velocity profiles, hydrostatic assumption and velocity direction along the slope. The question is as to whether similar reduced friction should be put into other types of models to reproduce observed runout of large landslides.

In this work, we investigate empirically the value of the grain–grain friction coefficient needed to reproduce landslide deposits using discrete element modelling. Small (down to laboratory scale) to huge events on Earth and on Mars will be simulated. Discrete element models (DEM) are more and more used to simulate natural landslides (e.g. Campbell 1989; Taboada and Estrada 2009; Lin and Lin 2015; Zhao et al. 2016, 2017; Feng et al. 2017), even though they also have strong limits that are however different from those of depth-averaged thin layer models. In particular, the size of the particles in the DEM simulations is much bigger than the size of the real grains involved due to related high computational cost. Furthermore, the real size distribution of the particles is generally unknown, and most DEM models do not take into account fragmentation processes that play a key role in natural landslides, in particular by changing the particle size during landslide propagation (Charrière et al. 2016; Zhao et al. 2017, 2018). As a result, the first issue is how the simulated runout from DEM is affected by the number (i.e. size) of grains within a given volume.

Before simulating natural landslides on simplified topographies, we first calibrate the model on a given set of laboratory experiments of granular flows. Then we test the model ability to reproduce the sensitivity of the deposit on the characteristics of the initially released mass (volume and aspect ratio *a* defined as its width divided by its height). We choose the widely studied granular collapse experiments. Laboratory experiments and numerical simulations have shown that the normalised runout distance of a dry granular mass spreading on a horizontal plane is controlled by the initial aspect ratio *a* of the mass (width divided by initial height) and that the actual volume of the column was unimportant (e.g. Lube et al. 2004; Mangeney-Castelnau et al. 2005; Kerswell 2005). On the contrary, experiments by Farin et al. (2014) showed that on steeper slopes, with angle above about half the repose angle of the material used, the normalised runout distance and other power laws depend not only on the aspect ratio but also on the landslide volume. Reproducing this behaviour is critical for numerical models of granular flows and has never been investigated numerically.

We first present the model and setup. We then perform a series of 3D DEM simulations of axisymmetric and dam-break rectangular collapse of granular columns on horizontal and sloping beds to calibrate the model and reproduce quantitatively the experimental deposit. We also investigate the minimum number of grains required for the results to be roughly independent of this number for a given volume. Keeping the values of the parameters defined in the calibration step, we compare simulated and measured sensitivity of the deposit to the volume and aspect ratio of the initial mass. Finally, we use field data to define the initial volume and mean slope of a series of natural landslides on Earth, on Mars, and also on Io and Iapetus. Using the simplified shape of the released mass and of the topography (inclined plane), we identify the value of the friction coefficient that makes it possible to best fit the observed runout distance of each landslide. Our results show that lower grain–grain friction is necessary to reproduce the runout distance of larger landslides, in agreement with what was found previously using depth-averaged continuum models. As a result, the empirical low friction needed to reproduce large landslide dynamics numerically is not related to the use of depth-averaged models, and it seems to be more generally required (Agliardi and Crosta 2003; Tang et al. 2009; Salciarini et al. 2010).

## Method and setup

### Contact force model and algorithm

Three-dimensional simulations of granular collapses are performed using molecular dynamics (MD), which consists in integrating the equations of motion of the grains over time (Cundall and Strack 1979). Such a method requires the modelling of the interaction forces to which the grains are submitted. The DEM code MODY-GS (Richard et al. 2008) is used. Each grain is represented by a non-deformable spherical object of uniform material. Deformations are taken into account by the contact model, which links the normal force acting on each grain to the overlap that occurs between the non-deformed spheres when the grain centres are closer than their diameters would allow.

The spheres interact only on contact through a classical linear spring–dashpot interaction law in the normal and tangential directions to their lines of centres (Fig. 2). The overlap *o* between two contacting spheres *i* (diameter *d*_*i*_) and *j* (diameter *d*_*j*_) positioned at ***r***_*i*_ and ***r***_*j*_ is defined by

1 $$ o=\left|{\boldsymbol{r}}_{ij}\right|-\left({d}_i+{d}_j\right)/2 $$

where *r*_*ij*_ = *r*_*i*_ − *r*_*j*_.

Following Shäfer et al. (1996), the normal and tangential contact forces are, respectively, given by

2 $$ {F}_{\mathrm{n}}={k}_{\mathrm{n}}\delta {n}_{ij}-{\gamma}_{\mathrm{n}}{v}_{\mathrm{n}} $$

and

3 $$ {F}_{\mathrm{t}}={k}_{\mathrm{t}}\Delta  {S}_{\mathrm{t}}-{\gamma}_{\mathrm{t}}{v}_{\mathrm{t}} $$

where ***n***_*ij*_ = ***r***_*ij*_/|***r***_*ij*_|, *v*_n_ and *v*_t_ are, respectively, the normal and tangential components of grain velocity and *k*_n, t_ and *γ*_n, t_ are, respectively, the elastic constant (i.e. the stiffness) of the springs and the damping constants of the dashpots. The nominal values of *k*_n_ and *k*_t_ used in this work are, respectively, 5.6 × 10^6^ and 2/7 *k*_n_. The value of *γ*_n_ is chosen such as the normal coefficient of restitution (see Shäfer et al. 1996) is equal to 0.88 and that of *γ*_t_ is set to 0. The two latter choices are commonly used in the literature (e.g. Silbert et al. 2001). Note that in dense granular flows, the effect of the restitution coefficient is weak due to the preponderance of multiple contacts (Dippel and Wolf 1999). *∆S*_t_ is the elastic tangential displacement between spheres, obtained by integrating tangential relative velocities during elastic deformation of the contact. The magnitude of *Δs*_t_ is truncated as necessary to satisfy a local Coulomb yield criterion *F*_t_ < *μF*_n_, where *F*_t_ = |*F*_t_|,*F*_n_ = |*F*_n_| and *μ* the Coulomb (i.e. sliding) friction coefficient. In the remainder of the paper, the grain–grain and grain–wall Coulomb friction coefficients will, respectively, be denoted *μ*_c_ and *μ*_w_. Obviously, frictionless spheres can be simulated simply by setting *μ* = 0. Finally, the position of the particles is updated according to the total forces and torques applied to them through contacts and the gravitational field. Rolling friction is accounted for by using the constant directional torque (CDT) model (Syed et al. 2017), which adds a torque, *T*_roll_ to the spheres. It depends on the relative shear angular velocity of the contact between two grain *ω*_shear_ = *ω*_*i*_ − *ω*_*j*_, where *ω*_*i*_ and *ω*_*j*_ are, respectively, the shear angular velocity of grain *i* and that of grain *j.* We use

4 $$ {T}_{\mathrm{roll}}=-{K}_{\mathrm{ms}}{R}_{\mathrm{eff}}{F}_{\mathrm{n}}{\omega}_{\mathrm{shear}}/\left|{\omega}_{\mathrm{shear}}\right| $$

where *R*_eff_ is the effective radius *d*_*i*_*d*_*j*_/(2*d*_*i*_ + 2*d*_*j*_) and *K*_ms_ is the rolling friction coefficient.

Although other more realistic models exist (Jiang et al. 2013; Syed et al. 2017), the CDT model is the simplest. Note, however, that it introduces a final residual kinetic energy which oscillates with a period equal to twice the time step adopted in the simulations (Ai et al. 2011) which may induce a slow creep. Such kind of oscillations is often observed in soft-sphere MD. They are the consequences of the visco-elastic nature of the force between grains and of the time discretisation. As any model, MD has limitations, yet we have checked that in our simulation, our results are not affected by the presence of these oscillations. In practice, we consider that the system is at rest if the ratio of kinetic energy to potential energy is lower that 10^−10^.

The general procedure used for modelling granular materials using DEM is the same for all types of geometry. The simulation starts with defining the boundary conditions such as walls and the physical properties of the particles. Then, the particles are “initialised”, i.e. their initial distinct position is defined as well as their initial velocity. Then, all the possible contacts between distinct objects are detected to determine the interaction force between the objects.

It is important to point out here that MD simulations are only a model and that, as any model, its validity range is limited. We do not claim to capture all the physical phenomena present in nature. The way dissipation is mimicked in the simulations is indeed oversimplified mainly because, in nature, it occurs at the (nano) scale of the grain surface asperities and not at the (much higher) natural length scale of DEM simulation: the grain size. We already discussed above the unperfected—but reasonable—way rolling friction is introduced in the simulations. In general, the way dissipation is taken into account in DEM is questionable. For example, Coulomb friction is an important source of dissipation in the simulation. Yet it is unclear if the same friction coefficient should be used for enduring and collisional contacts. Also, natural landslides are much more complex processes that involve various grain size and shapes, fragmentation and erosion during entrainment.

To illustrate this purpose, it has been demonstrated by impact experiments (Foerster et al. 1994) that, for glass beads, *μ* ~ 0.1 and this is independent of velocity. However, in DEM simulations of compact systems with enduring contacts, using *μ* = 0.1 results in insufficient dissipation. Consequently, for compact systems of glass particles, it is necessary to use *μ* > 0.3 to obtain realistic values of macroscopic mechanical properties (Bednarek et al. 2017) suggesting that the grain–grain friction coefficient has to depend on the nature of the contact (collisional, enduring) and thus on local grain neighbourhood. Addressing this point in DEM simulations is a complicated task since it requires knowing at the beginning of a contact if it will be collisional or enduring.

Thus, we choose the most simplified ways to mimic the different sources of dissipations (grain–grain and grain–wall (sliding friction, rolling friction) to model a physical system with a minimal number of input parameters being aware of the limited range of application of our approach.

### Numerical setup

**Fig. 3:**
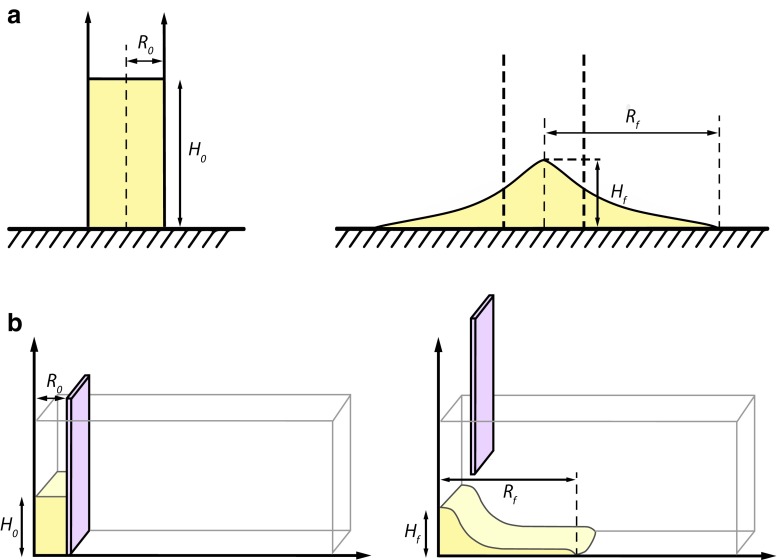
Numerical setup of granular column collapse model designed to simulate axisymmetric (**a**) and dam-break rectangular (**b**) collapses described in this paper: left initial state; right: final deposit state

The simulation setup is shown in Fig. 3. In the axisymmetric case, a cylindrical column of grains is prepared by deposing grains under the action of gravity until they reach a steady state. The initial column has length *R*_0_ and height *H*_0_ calculated in the downslope or normal direction, respectively. For simulations on inclined planes, the reference frame to calculate thickness, lengths and velocities is inclined along the slope. The inclined channel has some roughness imposed by particles glued to the plane. Furthermore, in the comparison with experiments, we take into account the erodible bed as in the experiments. The final length and height are *R*_f_ and *H*_f_, respectively. The column aspect ratio is defined as *a* = *H*_0_/*R*_0_. We also carried out numerical experiments on granular flow dam-break rectangular collapse. In this configuration, granular materials were initially deposited within a rectangular box resting on top of a channel with the same width as the granular column. After the confining box is quickly released, all the grains spread out along the channel. The number of particles in a given simulation can be varied in order to determine the minimum number required to describe landslide spreading appropriately. Times is counted dimensionless:

5 $$ \overset{\sim }{t}=t/\sqrt{H_0/g} $$

where *t* is time in seconds, *H*_0_ is initial particle pile height and *g* is the acceleration of gravity.

## Model calibration

Taking into account the aforementioned limits of our approach, we calibrate here the 3D DEM model on granular collapse experiments that have been widely studied on horizontal planes (e.g. Lube et al. 2004, 2005, 2007; Balmforth and Kerswell 2005), sloping beds (Mangeney et al. 2010; Lube et al. 2011; Farin et al. 2014), with grain/fluid mixtures, and with various particle shapes (Meruane et al. 2010; Rondon et al. 2011; Roche et al. 2011). Collapses have also been studied numerically using DEM (Calvetti et al. 2000; Staron and Hinch 2005, 2007; Zenit 2005; Lacaze et al. 2008; Girolami et al. 2012), shallow depth-averaged models (Mangeney-Castelnau et al. 2005; Kerswell 2005; Larrieu et al. 2006; Doyle et al. 2007; Hungr 2008; Lucas et al. 2011), and 2D visco-plastic or elasto-plastic models (Crosta et al. 2009, 2015; Lagrée et al. 2011; Ionescu et al. 2015). The DEM simulations were conducted in 2D (Staron and Hinch 2005; Zenit 2005) or in 3D (e.g. Girolami et al. 2012; Utili et al. 2015; Kermani et al. 2015). The DEM approach has been shown to be able to reproduce the general behaviour of unsteady collapses of a granular column and the key scaling laws observed experimentally on horizontal planes. The reported results qualitatively and sometimes quantitatively reproduce granular collapse experiments on horizontal slope and irregular topographies. However, to our knowledge, the question remains as to whether DEM can reproduce *quantitatively* granular collapse runouts and power laws on sloping beds and in particular the change in the dependency on the volume above a critical slope angle. Indeed, using continuum elasto-plastic or visco-plastic models, Crosta et al. (2015) failed to replicate the increase in runout observed in the presence of erodible layers on slope angle steeper than the critical slope angle. This is, however, an important issue for further modelling of real landslides or for representative experimental models and material characterisation.

### Parameter calibration and sensitivity analysis

We first calibrated the 3D DEM parameters on axisymmetric and dam-break experiments of granular collapse on a horizontal plane in a laterally confined condition.

The input physical parameters required for each simulation are the grain–grain (inter-particle) Coulomb friction coefficient (*μ*_c_), the particle–wall Coulomb friction coefficient (*μ*_w_), the coefficient of restitution (*E*_n_) and the coefficient of rolling friction (*K*_ms_). The coefficient of restitution depends on *k*_n_ and *γ*_n_ (Richard et al. 2008).

The grain–grain friction characterises a material and controls the flow properties.

Calibration of *μ*_c_ and *μ*_w_ was based on the range of values commonly used in previous analogue simulations (Lacaze et al. 2008; Staron and Hinch 2007; Girolami et al. 2012) and on comparisons with several experiments from Mangeney-Castelnau et al. (2005), Mangeney et al. (2010) and Farin et al. (2014). From calibration runs, we fixed *μ*_c_ = 0.45 and *μ*_w_ = 0.5, corresponding to friction angles *δ*_c_ = 24.2^°^ and *δ*_w_ = 26.5^°^, respectively. Note that the particle–wall friction angle is very large, even though it agrees with the value used by Girolami et al. (2012). The resulting values of *μ*_c_ and *μ*_w_ (0.45 and 0.5, respectively) remained constant for all the simulations presented in Figs. 5, 6, 7, 8, 9, 10 and 11.

To compare the general behaviour of our model with other DEM, we explored its sensitivity to the parameters involved, as was done in previous studies. We repeated many simulations with grain–grain friction values ranging from 0.05 to 1 in order to determine how these values affect landslide propagation. For these sensitivity tests, we choose arbitrarily a dam-break configuration with a slope angle *θ* = 0.1, *H*_0_ = 225 mm and *R*_0_ = 140 mm so that *a* ~ 0.8.

**Fig. 4:**
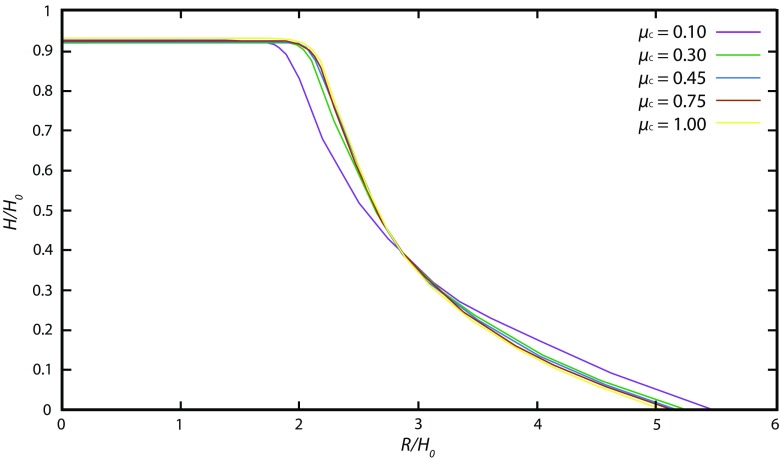
Influence of grain–grain friction on the deposit of granular material. Dam-break geometry, aspect ratio *a* = 0.85, initial length *R*_0_ = 82.5 mm, grain–wall friction coefficient for simulations *μ*_w_ = 0.45, coefficient of rolling resistance *K*_ms_ = 0.133

**Fig. 5:**
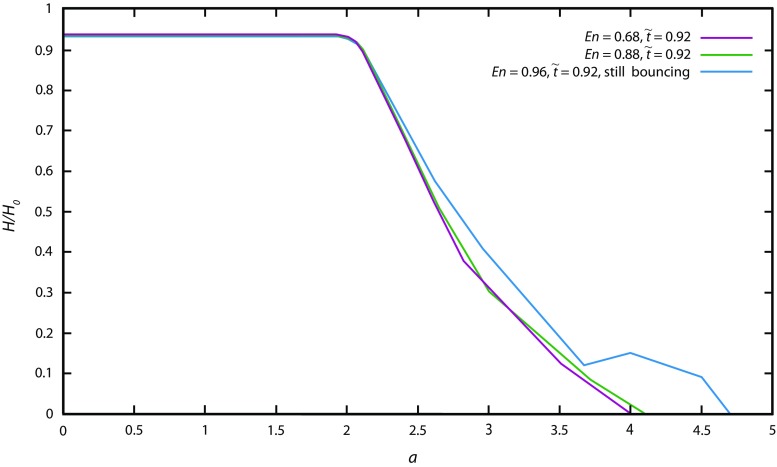
Influence of value of coefficient of restitution on the behaviour of granular material. Dam-break geometry case, *a* = 0.64, *R*_0_ = 60.5 mm, *μ*_c_ = *μ*_w_*μ*_c_ = 0.45, *K*_ms_ = 0.133

The results show that above a grain–grain friction coefficient value *μ*_c_ = tan *δ*_c_ = 0.3 (*δ*_c_ = 16.7^°^), the dynamics of spreading on horizontal planes and the final deposit does not change significantly when increasing *μ*_c_ (Fig. 4). We also varied the coefficient of restitution, which is a key parameter in the microscopic interaction model. Specifically, it controls the inelasticity of collisions and potentially could influence the rate of energy dissipation during the granular collapse. In agreement with former studies, we found that only very high restitution values (*E*_n_ → 1) change the overall spreading dynamics and lead to a larger runout distance (Fig. 5). As found by Staron and Hinch (2005) and Cleary and Frank (2006), we observe that its influence is negligible on the characteristics of the final static pile, except for the extreme case in which *E*_n_ = 1. In this study, this value is set to *E*_n_ = 0.88.

With particles free to roll, the mobility of the granular assembly is significantly higher than what is observed experimentally. Rolling friction takes into account that the surfaces of colliding particles and walls slightly deform. It is a small but potentially important force in runout scenarios. In real granular systems, resistance mechanisms may have different micro-mechanical origins, such as adhesion on the contact area, or steric effect due to surface roughness or non-sphericity about the contact point. We also chose to fix *K*_ms_ = 0.133 for the grain–wall and grain–grain contacts, which is in good agreement with the values tested by Ai et al. (2011) and Girolami et al. (2012).

Table 1 shows the values of micromechanical parameters used in earlier works and in this study.

**Table 1:** Summary of the physical parameters used in the literature and in this paper

Article	Number of grains, *N*	2D/3D discrete-element simulation	Diameter, *d* (mm)	Stiffness of the normal spring, *k*_n_	Grain–grain friction, *μ*_c_	Grain–wall friction, *μ*_w_	Coefficient of restitution, *E*_n_	Aspect ratio, *a*	Coefficient of rolling friction for particle/wall contacts, *K*_ms_
Zenit (2005)	100–100,000	2D	3.5	7 × 10^5^	0.3 and 0.57		0.75	0.3–12	
Staron and Hinch (2005)	1000–8000	2D			1		0.5	0.2–17	
Cleary and Frank (2006)	165,000	2D	1.9–2.1		0.3		0.4	1.91	
Brodu et al. (2013)	11,000	3D	2.97	2 × 10^5^	0.33		0.972	0.1	0.133
Girolami et al. (2012)	3000–15,000	3D	3/5	4 × 10^5^	0.5	0.5	0.75	0.5–18	0.133
Lacaze et al. (2008)	1600–6000	2D	2.5 and 5		0.15 and 0.35	0.35	0.5	01.0–2.24	
Lo et al. (2009)	1000–1500	2D	1 and 2	4 × 10^6^	0.577		0.2	0.2–10	
This study	200–8000	3D	3.2–16.5	4.5 × 10^5^	0.1–0.58	0.1–0.58	0.88	0.05–1.6	0.133

### Detailed comparison with laboratory experiments

**Fig. 6:**
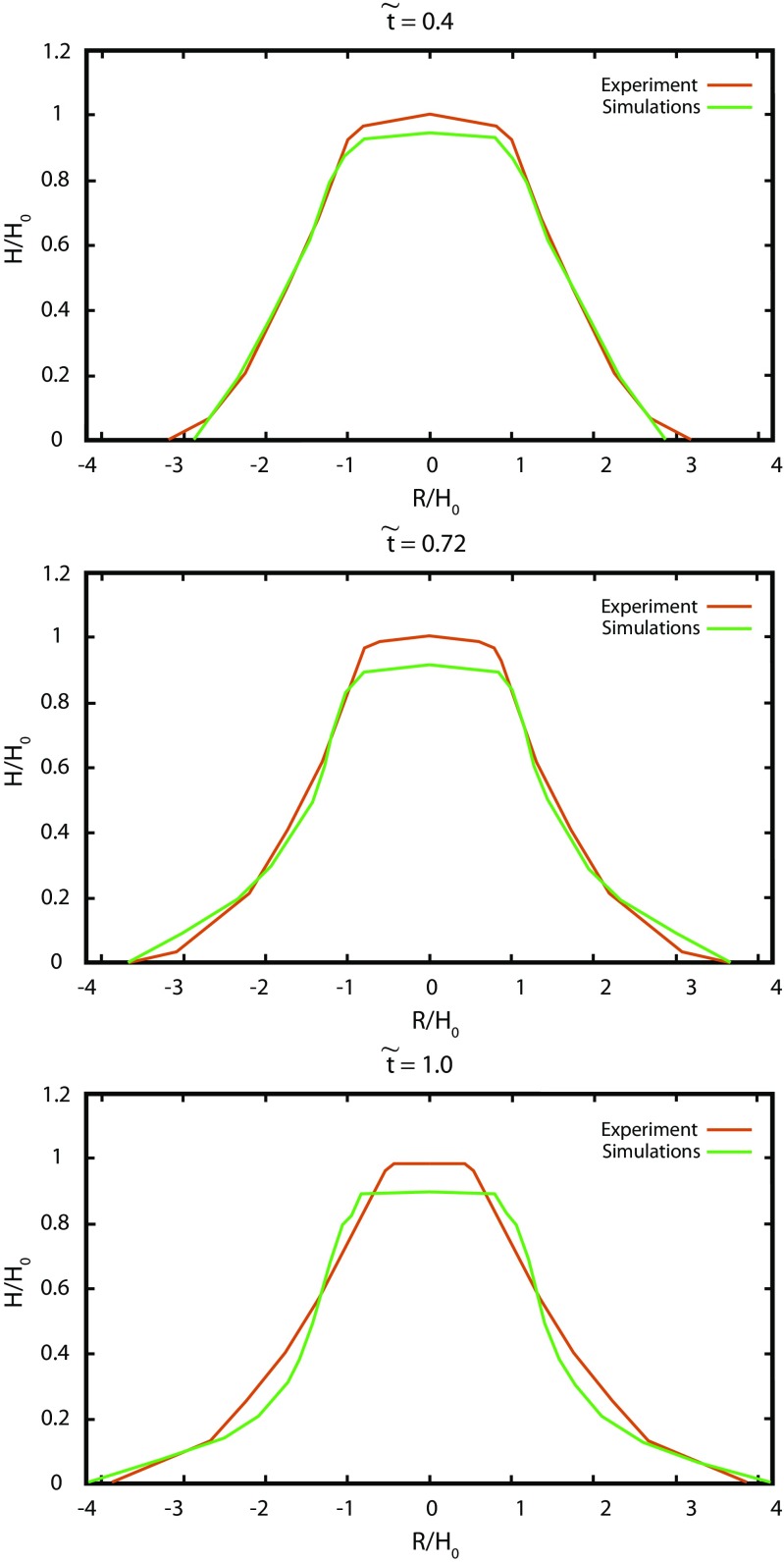
Comparison between cross sections along a plane cutting the pile going through the centre of the column during the spreading of an axisymmetric granular mass at different normalised time; *a* = 0.8, *R*_0_ = 70.5 mm, for laboratory experiments (after Mangeney-Castelnau et al. 2005) and DEM simulations. In this figure and the next ones, grain–grain and grain/wall Coulomb friction coefficients for simulations *μ*_c_ = *μ*_w_ = 0.45, the coefficient of restitution *E*_n_ is 0.88, and the coefficient of rolling resistance *K*_ms_ is 0.133

**Fig. 7:**
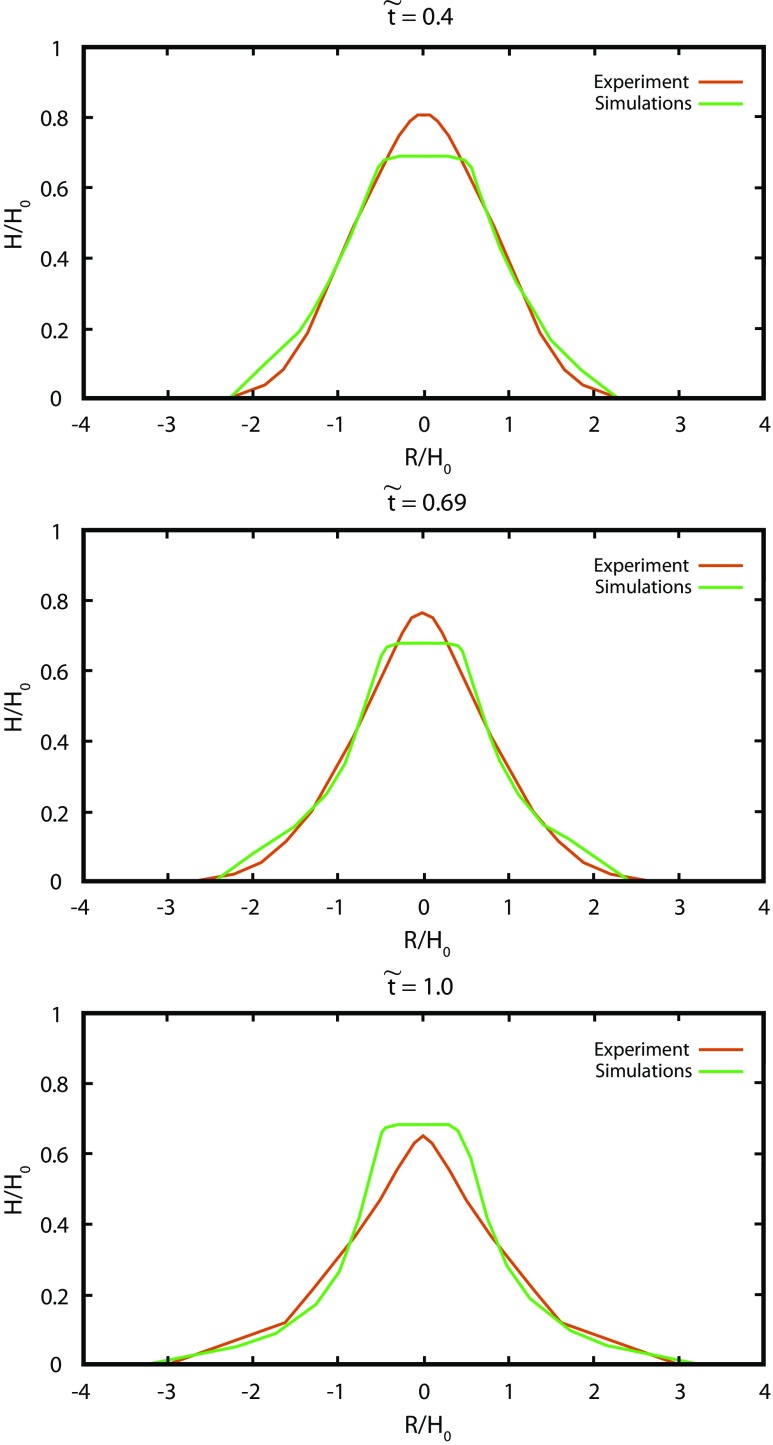
Comparison between cross sections along a plane cutting the pile going through the centre of the column during the spreading of an axisymmetric granular mass at different normalised time; *a* = 0.56, *R*_0_ = 70.5 mm, for laboratory experiments (after Mangeney-Castelnau et al. 2005) and DEM simulations. Other parameters as in Fig. 6

Let us first compare 3D DEM simulations with axisymmetric granular collapses of small aspect ratios, *a* < 1 presented in Mangeney-Castelnau et al. (2005). The numerical experiments have been performed with the following initial geometries: for *a* = 0.56: *R*_0_ = 70.5 mm and *d* = 5.6 mm; for *a* = 0.8: *R*_0_ = 70.5 mm and *d* = 6.3 mm, where *R*_0_ is a radius of cylindrical column and *d* is a grain diameter. Note that in the experiments, the grain size is about 10 times smaller, i.e. *d* = 0.35 mm. The axisymmetric collapse of the granular mass with time, measured by laboratory experiments, is well reproduced by the numerical simulations for the initial aspect ratio *a* = 0.56 (Fig. 6) and *a* = 0.8 (Fig. 7). The main difference between the granular collapse experiments on the horizontal plane and the DEM modelling is observed at the top of the pile, whereas the front region is quite similar. This disagreement could be due to the different initial packing of grains (Kermani et al. 2015).

**Fig. 8:**
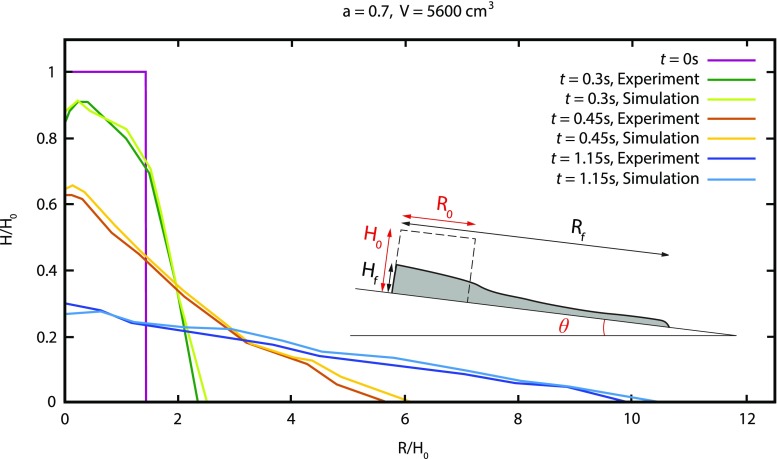
Comparison between the experimental and numerical thickness profiles during the spreading of an initially rectangular granular mass over an inclined rigid bed (*θ* = 22°), with *μ*_c_ = 0.45, *μ*_w_ = 0.5, *a* = 0.7 and volume *V* = 5600 cm^3^. The numerical simulations are compared with the experiments from Farin et al. (2014). The inset relates the parameters in this figure with their definition as from Fig. 3

We now investigate the spreading and deposit of a dam-break column on an inclined bed confined between frictional walls. Numerical simulations were conducted in an identical configuration to that used in the experiments conducted by Farin et al. (2014). These experiments were carried out on a rigid, rough channel for slope angles ranging from *θ* = 0° to 24° and volumes from *V* = 1.400 to 12.600 cm^3^, with grain diameter *d* = 7 mm (*d* = 0.6–0.8 mm in laboratory experiments). Aspect ratios *a* ranged from *a* = 0.3 to 1.24, consistent with many geophysical flows. Figure 8 shows that the laboratory experiments of the spreading of granular mass are well reproduced by DEM simulations until the arrest of the spreading front. Interestingly, the DEM well reproduces the dilation of the mass at *t* = 0.45 s (i.e. the volume is the same in the simulation and in the experiments). This is obviously not the case when using incompressible continuum visco-plastic models where the flowing mass has a smaller volume than the experimental mass. The difference between the simulation and experimental results could be due to the removal of the gate at the initial instant in the experiments that is not taken into account in our simulations. It has, however, an impact on the spreading dynamics even though it does not change the deposit (Ionescu et al. 2015). Indeed, the presence of the gate leads to front positions about 10–15% smaller than when there is no gate, which is in agreement with what we observe here (see Figures 14 and 15 of Ionescu et al. 2015).

### Insight into the flow structure

**Fig. 9:**
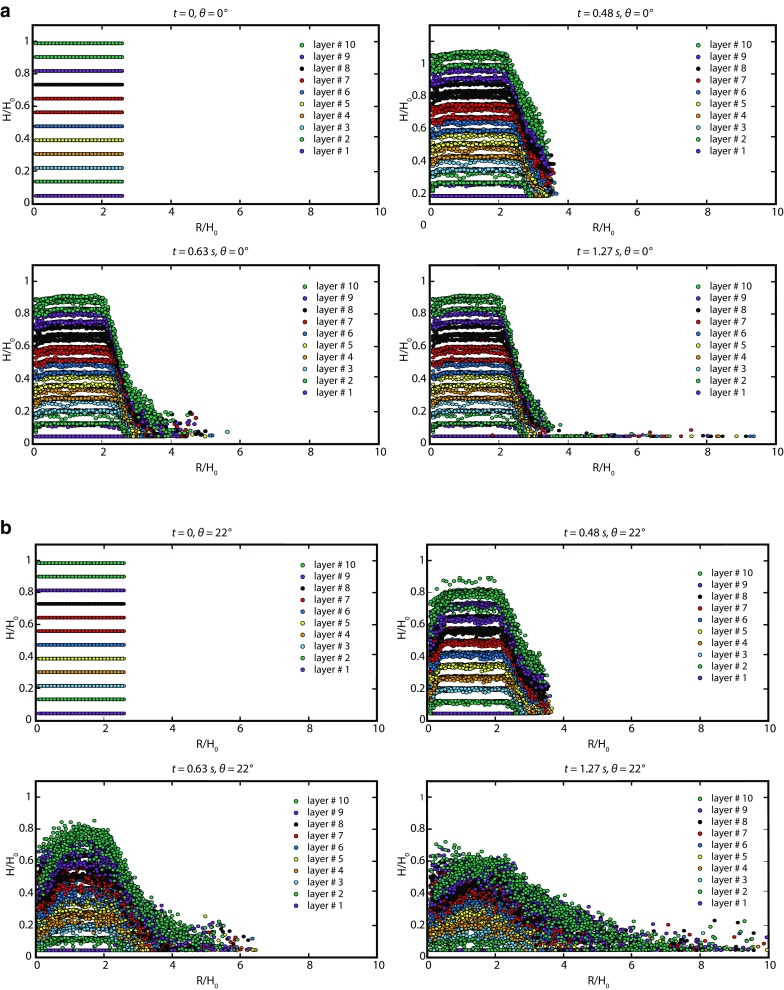
**a** Deformation of granular assemblage during the simulation in the dam-break rectangular case (Fig. 3b) and a horizontal bed; *a* = 0.7, *V* = 8750 cm^3^; *μ*_c_ = 0.45, *μ*_w_ = 0.5, *K*_ms_ = 0.133. The latest time corresponds to the end of the granular motion. The profiles are taken at mid-distance from the two dam walls. **b** Deformation of granular assemblage during the simulation in the dam-break rectangular case and a sloping bed; bed slope *θ* = 22°, *a* = 0.7, *V* = 8750 cm^3^, *μ*_c_ = 0.45, *μ*_w_ = 0.5, *K*_ms_ = 0.133. The latest time corresponds to the end of the granular motion. The profiles are taken at mid-distance from the two dam walls

Figure 9 shows snapshots of the deformation of granular assemblies with the whole granular column divided into 10 equal-sized horizontal layers with different colours. Simulations with marker beads also indicate that these granular flows do not involve much mixing between the initially horizontal layers, which become deformed and distorted but not mixed during their displacement, as noted experimentally by Lube et al. (2004). The grains initially located in the uppermost region end up blanketing most of the final pile.

In the horizontal bed case (Fig. 9a), the upper surface of the released material is divided into an inner stationary zone (which remains at the initial height) outside of which the material is flowing. We see that only the frontal part of the flowing layer is in contact with the basal surface, whereas the major flow takes place over the deposited material. Much of the upper free surface remains undeformed; the flow simply consists of the fall of the edges of the initial column. By contrast, the grains localised inside the column have no motion and play no role at all in the spreading; i.e. only a small part of the initial volume is mobilised. But in the sloping bed case (Fig. 9b, i.e. the base wall is inclined), a significant amount of particles is involved in the flow, in agreement with what was found in continuum models (Ionescu et al. 2015; Crosta et al. 2015). Note that due to confinement, the final heap is relatively steep (Metayer et al. 2010 and references therein).

### Calibration of the number of grains necessary to obtain stable results

**Fig. 10:**
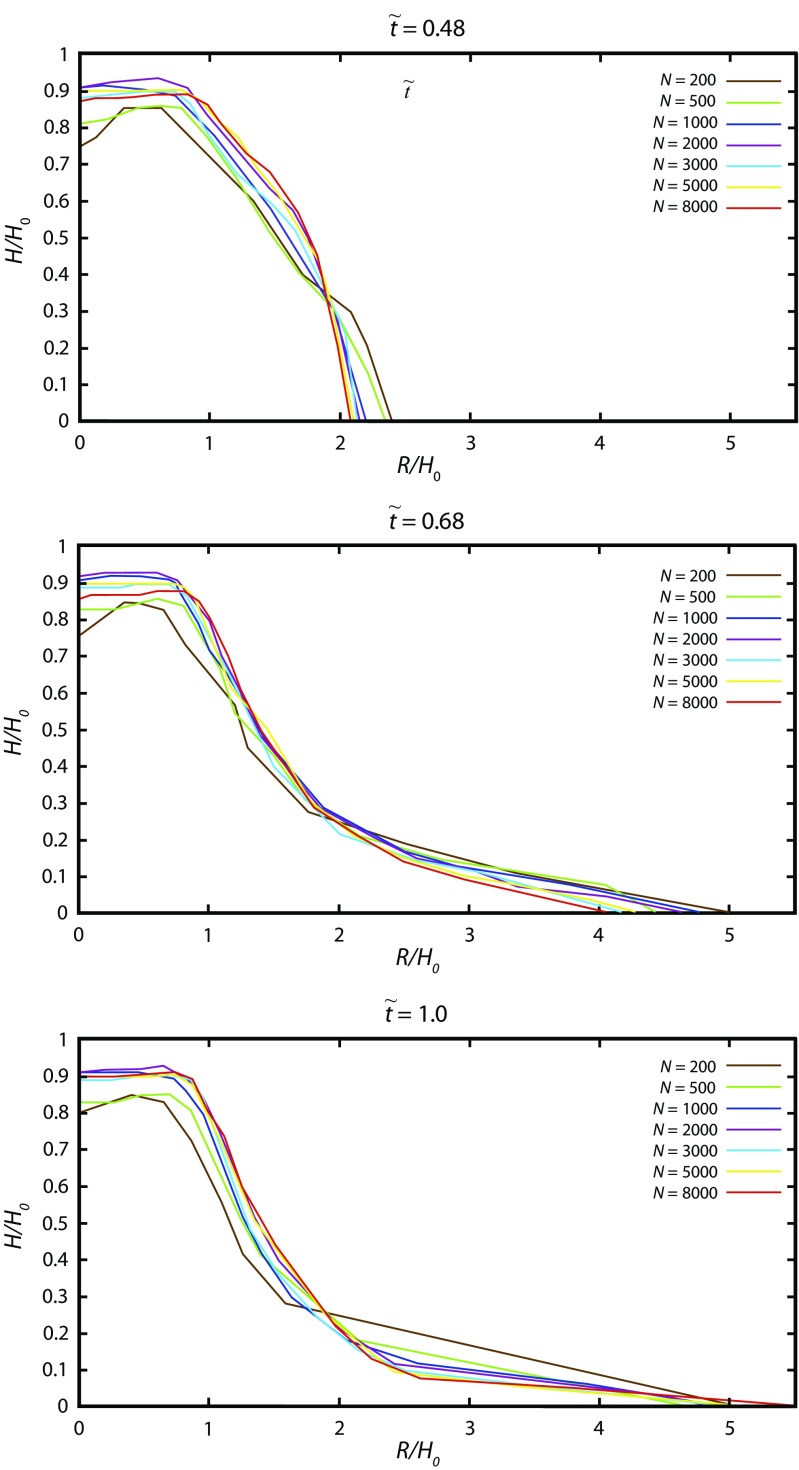
Influence of the number of grains (*N*) on the thickness profiles at different times for the dam-break case (Fig. 3b) with *a* = 1.24, *V* = 5600 cm^3^, *μ*_c_ = 0.45, *μ*_w_ = 0.5, *K*_ms_ = 0.133

The simulation time rapidly increases when increasing the number of grains. In order to use DEM as an empirical tool to simulate real geophysical flows, the simulated deposit should not depend drastically on the number of grains for a given volume. Indeed, as discussed above, the real distribution of grains involves much smaller grains than the one possible to use in DEM simulations with a reasonable computational cost. In order to investigate the sensitivity of DEM simulations to the number of grains involved, we conducted simulations for the dam-break rectangular case with different numbers of particles ranging from 200 to 8000 (Fig. 10). When the number of grains is higher than 1000, the particle number does not significantly change the basic behaviour of the granular material and the results obtained with 5000 and 8000 grains are very close, with stabilisation of profile shapes occurring at 8000 whatever the set of mechanical properties. This observation suggests that, even if the size of the particle is much larger than real particles with respect to the number of grains, the DEM simulations performed in this work may be used to estimate the basic behaviour of large-scale geophysical flows. In the following simulations, 8000 particles are taken.

### Sensitivity of runout on initial volume and aspect ratio

To our knowledge, we investigate here for the first time with DEM the sensitivity of the runout distance on the volume *V* and aspect ratio *a* for inclined granular flows. In particular, the question is as to whether DEM reproduces the increase in normalised runout distance with volume above a critical slope angle *θ*_c_ as observed experimentally by Farin et al. (2014).

We simulate experimental collapse of rectangular columns confined in a channel performed by Farin et al. (2014). Analytical solutions provide insights into the scaling laws observed experimentally and numerically (Kerswell 2005; Mangeney et al. 2010). Following Lube et al. (2005) and Kerswell (2005), we define the runout distance as the change in pile length *R*_n_ along the slope, normalised by the initial length *R*_0_:

6 $$ {R}_{\mathrm{n}}=\left({R}_{\mathrm{f}}-{R}_0\right)/{R}_0 $$

For granular collapses over sloping bed with a slope *θ* < *θ*_c_, where *θ*_c_ is the critical angle above which *R*_n_ depends on the volume involved, the normalised runout distance obeys the empirical relation (Mangeney et al. 2010; Lucas et al. 2014, Eq. 10 of their Supplementary Note 2):

7 $$ {R}_{\mathrm{n}}= ka/\left(\tan \delta -\tan \theta \right) $$

with *k* = 1 and tan*δ* is the effective friction coefficient. When *θ* > *θ*_c_, *R*_n_ not only depends on *a* but also depends on the volume involved (Figures 3 and 4 of Farin et al. 2014).

**Fig. 11:**
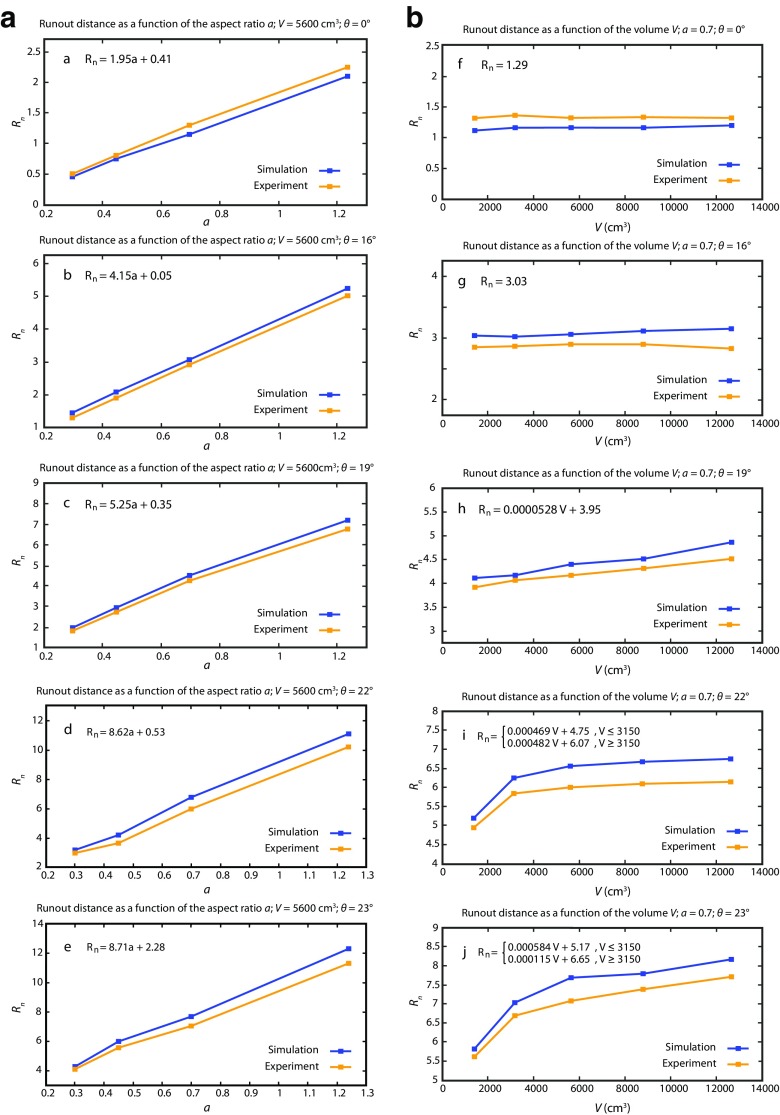
Part 1**.** Influence of aspect ratios *a* and volumes *V* on the normalised runout distance for the dam-break configuration and horizontal bed (*θ* = 0°), and bed sloping *θ* = 16°, *θ* = 19°, *θ* = 22° and *θ* = 23°, for constant *V* = 5600 cm^3^; *μ*_c_ = 0.45, *μ*_w_ = 0.5, *K*_ms_ = 0.133. The numerical results are plotted together with the experimental results obtained by Farin et al. (2014). The obtained linear fits are given for each plot. Part 2**.** Influence of aspect ratios *a* and volumes *V* on the normalised runout distance for the dam-break configuration and bed sloping *θ* = 16°, *θ* = 19°, *θ* = 22° and *θ* = 23°, for constant *a* = 0.7; *μ*_c_ = 0.45, *μ*_w_ = 0.5, *K*_ms_ = 0.133. The numerical results are plotted together with the experimental results obtained by Farin et al. (2014). The obtained linear fits are given for each plot

Series of simulations with different aspect ratios *a* and initial volumes *V* for slope angles *θ* = 0^°^, 16^°^, 19^°^, 22^°^ and 23^°^ slope angles were performed (Fig. 11). For each simulation, the final distance *R*_f_ is evaluated from the position of the grains connected to the main mass by at least one contact. The general trend obtained in the simulations is consistent with the experimental trend obtained by Farin et al. (2014). For all slope angles, the runout distance is proportional to the aspect ratio (Fig. 11(a–e)) for a given volume. For *θ* = 0^°^ to *θ* = 16^°^, the runout distance does not depend on the volume involved (Fig. 11(f, g)). At *θ* = 19^°^, volume dependency is clear although not strong and in very good agreement with experimental data (Fig. 11(h)). At *θ* = 22^°^ and *θ* = 23^°^, the runout distance markedly depends on the volume (Fig. 11(i, j)), with a dependency that is very strong for the smallest volumes and that decreases with increasing volume, leading, for *V* > 3150 cm^3^, to an almost linear relation between runout distance and volume.

## Empirical friction derived from landslide runout simulation

Our model has been shown to reproduce a wide range of laboratory experiments without changing the model parameters and to reproduce quantitatively the sensitivity of the deposit to initial volume and aspect ratio of the released mass under confined conditions. Based on this result, we apply the 3D DEM to simulate simple configurations with characteristic dimensions (volume, aspect ratio) close to natural landslides.

We perform 3D DEM simulations with characteristics close to natural landslides. The natural landslides are simulated using largely simplified DEM, with the spreading mass flowing on a slope of constant angle *θ* corresponding to the mean sliding slope, and for reconstructed volume *V* and aspect ratio *a*. The slope angle that follows the main direction of observed landslide mass motion *θ* is calculated from field data (in the case of terrestrial landslides) or remote sensing analysis (for Martian landslides) reported in earlier publications (see parameter values in Supplementary Table 1 and the corresponding reference list in Supplementary Table 2). For a small number of landslides (three on Mars and two terrestrial), the required information was not clear or not existing, and we determined the parameter values ourselves using combined photointerpretation at very high resolution (image resolution > 1 m/pixel) of landslide scars, propagation direction and maximum runout distance, with the help of digital topography at the appropriate scale (MOLA tracks for Mars; ASTER GDEM and SRTM3 for Earth). A granular column of volume *V* = *WH*_0_*R*_0_, where *W* is landslide width, is released on the inclined plane of slope *θ*. We choose *W* = *H*_0_ so that $V={H}_0^2{R}_0$**.** The setup is a dam-break configuration with lateral walls separated by the distance *W*. For all the simulations, we take 8000 grains, which would correspond to grain diameter of *d* = 10–50 m for real volumes of 10^7^–10^9^ m^3^ for example. We take the same value for the grain–grain (*μ*_c_) and grain–wall (*μ*_w_) friction coefficients (Supplementary Table 1, column 4). For each landslide, this friction coefficient is adjusted in order to match the normalised runout distance (column 3) observed in the field or on image and topography data (see the list of used references in Supplementary Table 2). For a given landslide, by successive attempts, we gradually narrowed the range of possible friction coefficients in the simulations that matches the observed large-scale runout distance. This exact distance was however rarely obtained in the simulations because the stopping point of the few individual grains ahead of the main sliding body is not predictable.

**Fig. 12:**
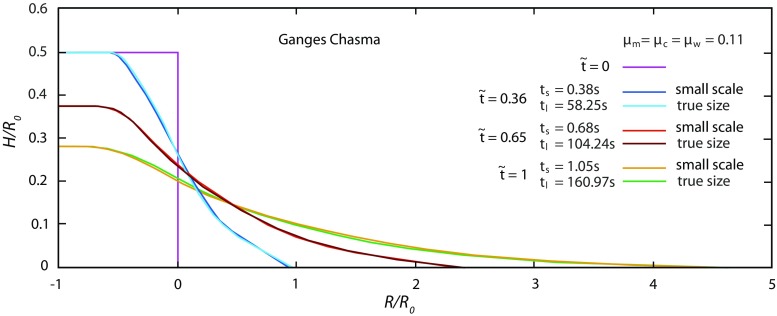
Comparison between runout distances for small-scale simulations (*R*_0_ = 800 mm)and large-scale (true landslide dimensions) simulations for the Ganges Chasma 1 landslide (*R*_0_ = 6580 m). In both cases, the same runout distance is obtained for the same number of grains (8000), aspect ratio (initially 0.5), friction (*μ*_c_ = *μ*_w_ = 0.11) and bed slope (*θ* = 1.1°)

**Fig. 13:**
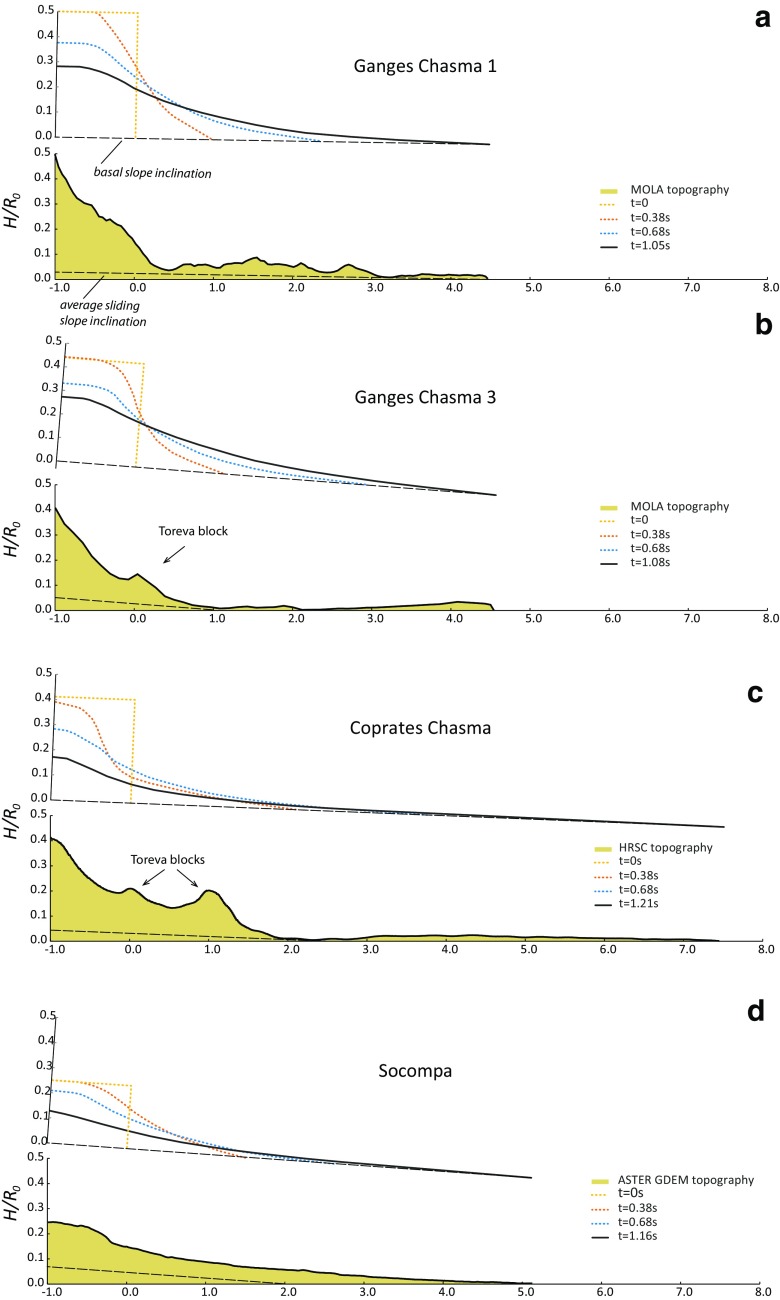
**a**–**d** Comparison between normalised runout versus normalised height for real landslides (black) and simulated landslide deposits (coloured). All the simulations were conducted using the dam-break configuration. For terrestrial landslides, the topography data are either from SRTM (ground spacing 90 m) or ASTER GDEM (ground spacing 15 m). For Martian landslides, the topography data are either from Mars Global Surveyor/MOLA or, when available, from the Mars Express/HRSC-derived Valles Marineris digital elevation model (Gwinner et al. 2009). The data used in the simulations are provided in Supplementary Table 1. The mismatch between topography from simulations and from natural landslide is inherent to the difference between natural scar geometry and dam-break geometry and lateral spreading of deposits (perpendicular to the profiles) in natural landslides, which is not allowed in the simulations, where the grains are laterally bounded by walls, from Toreva blocks, and from the influence of uneven basal plane topography

Using small-scale simulations, like in the calibration tests, or large grains to simulate true landslide dimensions does not significantly affect the predicted normalised landslide propagation distance for similar aspect ratio and friction, as illustrated for one landslide in Fig. 12. By way of illustration, simulated profiles of some landslide deposits are compared with real topographic profiles in Fig. 13. For each landslide, the true topographic profile is given in Fig. 13 without topographic correction. Simulated profiles are provided above each true landslide profile. Although the simulations take the observed slipping plane slope into account (as from Supplementary Table 1), the results of the simulations are provided in a coordinate system which is not rotated. For this reason, the simulation profiles in Fig. 13 are rotated by an amount equal to the slipping plane angle so as to compare with the true landslide profiles. Coordinate distortions between the natural and simulated profiles do not make possible a point by point correspondence, but the general geometries can be compared. The agreement between the topography of the simulated profiles and the topography of natural landslides depends on block size homogeneity in the landslide, as well as the discrepancy between the natural basal topography and the averaged inclined topography used in the simulations. For instance, the Ganges Chasma 3 and Coprates Chasma landslides (Fig. 13b, c) have Toreva blocks along the landslide profile (see Fig. 1 for the Coprates Chasma landslide), which concentrate a significant portion of the landslide volume that the DEM approach cannot reproduce. On the contrary, Toreva blocks of the Socompa landslide (Fig. 1) are not located along the landslide profile, significantly improving the match between the natural and simulated profiles (Fig. 13d).

**Fig. 14:**
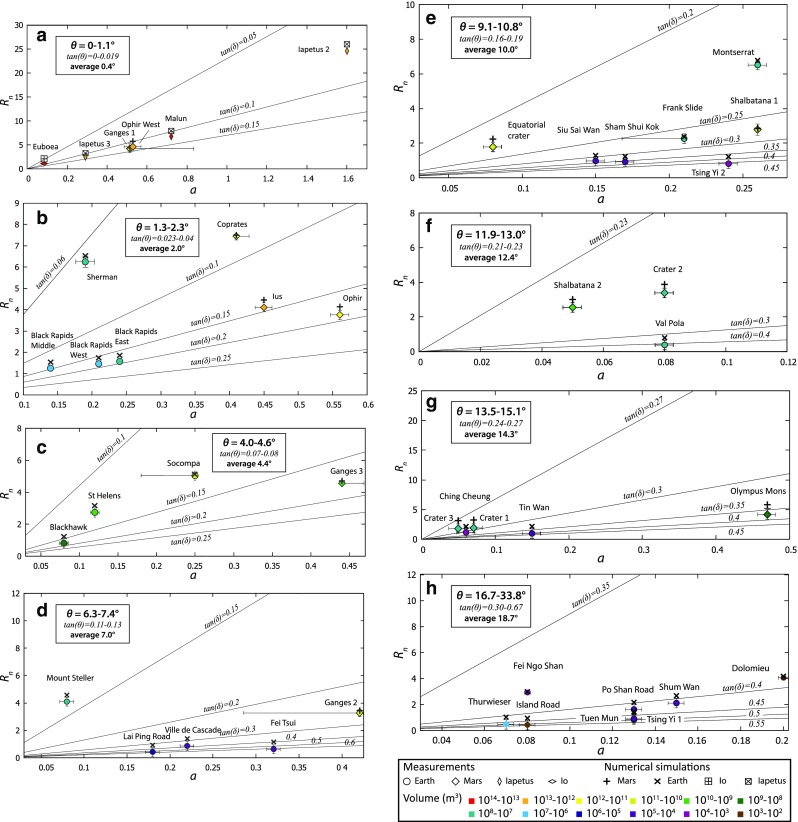
**a**–**h** Normalised runout as a function of aspect ratio for landslides of similar slopes on Mars and Earth, as well as for three landslides in the outer solar system. The coloured symbols are data from real landslides and the crosses and empty symbols are simulations results. The plain lines represent theoretical curves of constant slope and effective friction angle calculated from Eq. (7) using data in Supplementary Table 1. The data points are from this work and from earlier publications (see Supplementary Table 2). For the Ganges 1, Ganges 2, Ganges 3 and Coprates landslides of Mars as well as the Frank and Socompa landslides, two possible aspect ratios *a* were identified based on two different interpretations of orbital imagery. One was selected as the most likely correct and gives the location of the data point. The other possibility is at the end of the horizontal error bar. The extent of the deposits is well constrained by imagery, resulting in very small error on the runout distance *R*_n_

The runout distances obtained from small-scale DEM simulations and the scaling laws obtained in Fig. 11 are quantitatively compared with natural landslides on Mars and Earth in Fig. 14. A few data on landslides on giant planet satellites (Lucas et al. 2014) are also available and are incorporated into the dataset. Figure 14 gives the normalised runout distance of natural and simulated landslides as a function of aspect ratio. Each landslide has a distinct slope angle. To distinguish the effect of the slope and of the friction coefficient, distinct plots were generated, each of them for a given slope range (information on how the slope classes were determined is in Supplementary Fig. 1). Each graph also shows isolines of effective friction derived from Eq. (7); the friction coefficients obtained for each simulated landslide are reported in Supplementary Table 1.

The simulated runouts (empty symbols, see inset) systematically plot a little higher than the runout of natural landslides (filled symbols); this is a consequence of the unpredictable stopping point of the frontmost grain, as indicated earlier. The plotted simulated runout and the corresponding friction coefficients correspond to the friction coefficient that allows the simulated runout of the frontmost grain to exceed the observed large-scale runout distance by the smallest distance possible. Obviously, narrowing the runout distance discrepancy between the simulated and natural, large-scale landslides would require additional parameters to be accounted for, such as the variations of sliding plane slope angles along and across the sliding direction, and also faces the limits of DEMs in taking real behaviour of the granular material involved into account.

For very gentle slopes (*θ* = 0.4^°^, Fig. 14a), the dataset includes only very voluminous landslides (10^11^ to 10^14^ m^3^, in red–orange–yellow–light green tones), including two on Mars, one on Io and three on Iapetus. Landslides of dimensions similar to the Martian landslides are very rare on Earth, and none developed on flat or nearly flat terrain. Very low friction is needed in DEM simulations to reproduce their runout, in agreement with the models based on a continuum approach (Lucas et al. 2014). Less voluminous landslides (< 10^10^ m^3^, darker tones) do never require such low frictions.

**Fig. 15:**
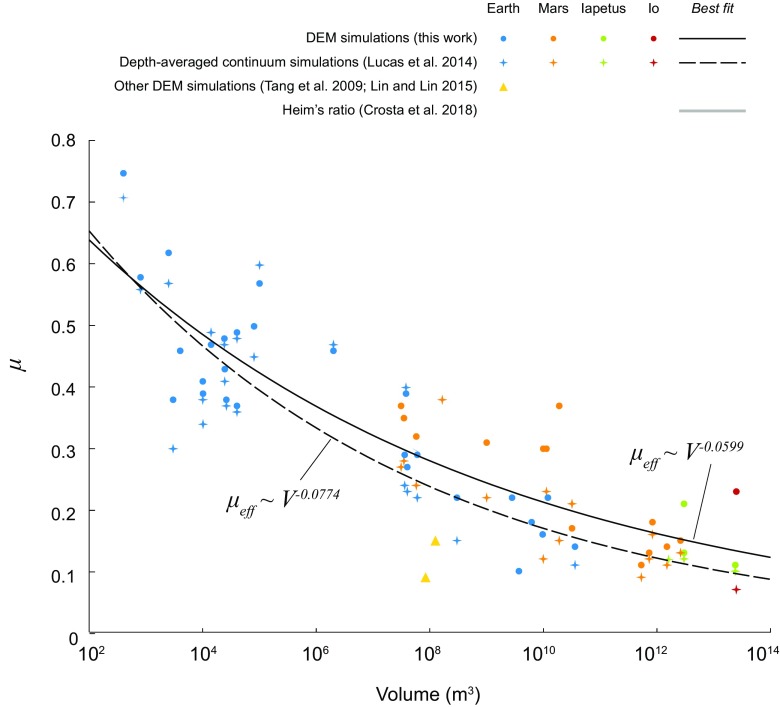
Microscopic (*μ*_m_ = *μ*_c_ = *μ*_w_) friction coefficient as a function of the volume of landslides in Fig. 12, compared with friction coefficients calibrated to reproduce real landslide runout using depth-averaged shallow continuum models obtained by Lucas et al. (2014, their Supplementary Table 1, corresponding to most of the dataset used here and given in our Supplementary Table 1), and the best friction obtained for DEM simulation of the 2009 Butangbunasi River landslide (Lin and Lin 2015, lower triangle) and the 1999 Tsaoling landslide (Tang et al. 2009, upper triangle). The best power-law fit and corresponding exponent from this work are given, together with the best power-law fit and exponent found by Lucas et al. (2014, Eq. 19 in their Supplementary Note 2)

As described above, for each landslide, we conducted simulation runs to calibrate the grain–grain and grain/wall friction coefficients *μ*_c_ (*μ*_w_ = *μ*_c_) (see Supplementary Table 1) that best reproduce the real runout distances (Fig. 13). We found that this empirical friction coefficient clearly decreases with increasing volume for volumes approximately larger than 10^3^ m^3^ (Fig. 15). The obtained friction coefficients are similar to the friction coefficients obtained by simulating the landslides with the depth-averaged continuum model SHALTOP (Lucas et al. 2014), although a little higher for similar volumes. With DEM, we obtain the following empirical fit (Fig. 15) for volumes larger than 10^3^ m^3^:

8 $$ {\mu}_{\mathrm{eff}\sim }\ {V}^{-0.0599} $$

which is very close to the fit determined by Lucas et al. (2014) using depth-averaged continuum models:

9 $$ {\mu}_{\mathrm{eff}}\sim {V}^{-0.0774} $$

## Discussion

The deposit simulated with DEM is shown to be approximately independent of the number of particles starting from 8000 particles in the simulation. This suggests that application of DEM to simulate natural landslides may not be too much affected by the mean size of the particles in the model (at least when considering monodisperse particles), that will be obviously much larger than most of the particles involved in the real event. However, factors such as particle shape, polydispersity, fragmentation and resulting segregation or energy dissipation effects, lateral expansion and water are expected to substantially affect the results; taking such factors into account would generate very high computational cost required to take into account the real distribution of sizes in natural landslides (e.g. Mead and Cleary 2015).

It is well established that landslide runout distance is proportional to aspect ratio on horizontal slopes, and laboratory experiments (Farin et al. 2014) showed that it is also proportional to landslide volume for slopes steeper than 16–19°. The systematic DEM simulations reported here (Fig. 11) agree with these results without having to change the value of the friction coefficient. As a result, with a constant microscopic friction coefficient *μ*_c_, we can reproduce the aspect ratio and volume dependency of granular flows at the laboratory scale over a large range of slopes without having to introduce any friction weakening effect.

On the contrary, when trying to reproduce the runout distance of natural landslides, it is necessary to empirically reduce the friction coefficient *μ*_c_ in DEM as the landslide volume increases as it was observed using shallow depth-averaged models. The grain–grain friction necessary to reproduce the runout of large landslides is very small (0.1–0.2), in agreement with the very small macroscopic effective frictions used in continuum depth-averaged models. Macroscopic friction results from a combination of grain–grain friction coefficient and geometrical arrangement between the grains. The geometrical arrangement only leads to macroscopic friction around 0.1, suggesting that in real landslides the real grain–grain friction is very small, which is confirmed by our DEM simulations.

This work, therefore, validates the view that volume (or velocity) dependency of friction is necessary to reproduce landslide propagation whatever the model used (DEM or continuum), and could therefore reflect a real physical behaviour. Note, however, that the DEM simulations here are performed in the dam-break configuration on simple inclined planes whereas depth-averaged simulations were performed on 3D complex topography with more accurate shape of the initially released mass. Numerical simulation using depth-averaged models, however, showed that the runout distance is a very robust parameter that is only weakly influenced by the geometry of the initial scar (Lucas et al. 2011). Lateral expansion of the deposit is also not allowed in the DEM simulations reported here, contrary to many natural landslides and continuum models. 3D simulations would be necessary to constrain the friction coefficient on the total extent of the deposit. However, Lucas et al. (2014) showed that using the friction coefficient constrained on the runout distance makes it possible to well reproduce the global deposit area (see figure 6 of their supplementary material). Interestingly, the grain–grain friction coefficient calibrated to reproduce the Tsaoling landslide (*V* ~ 126 million m^3^) using 2D DEM is *μ*_c_~0.15 (Tang et al. 2009), not so different to the effective friction coefficient calibrated with depth-averaged shallow models to reproduce the same landslide (*μ*_eff_~0.105) (Kuo et al. 2009). The grain–grain friction required to reproduce this landslide is higher than the macroscopic effective friction as observed in Fig. 15 when comparing empirical friction coefficients obtained from DEM to those obtained with shallow depth-averaged models. Including the presence of water would make the comparison even more complex.

Lucas et al. (2014) noted, using a depth-averaged continuum approach, that friction is inversely proportional to volume in a variety of environment conditions, from tropical wet (Taiwan) to glacial dry (Alaska); lithology, e.g. clays (Taiwan) to volcanic rocks (St Helens); size, from 10^2^ m^3^ (Island Road) to 10^13^ m^3^ (Euboea); and planetary body (Earth, Mars, Iapetus, Io), and Crosta et al. (2018) found a similar empirical correlation for a dataset of more than 200 Martian landslides. Using DEM, we also do not see clear differences between the different environments or planets in the general trend of friction weakening. This suggests, at the leading order, the existence of common mechanisms that lead to friction weakening whatever the planet and geological environment. Obviously, the local environment will modulate and possibly involve other physical processes, rheological laws and parameters that may explain the great dispersion of the data around the mean friction weakening empirical trend. For instance, as noted in the “Introduction”, landsliding on an icy bed on Mars, or sliding of a rock body that includes ice, may also contribute to friction weakening. However, very low friction is also inferred for landslides on bodies without ice (Io, Iapetus), suggesting that if ice plays a role in friction weakening, it may not play a critical role.

This friction weakening with volume can also be interpreted as velocity-dependent frictional weakening (Lucas et al. 2014; Liu et al. 2015). An intuitive explanation of this velocity weakening is that higher velocities increase the fluctuations in granular flows and may locally decrease the volume fraction, possibly decreasing frictional dissipation and enabling more complex flows. As explained in the previous section, Lucas et al. (2014) found a phenomenological relationship between the effective friction and the flow velocity by comparing numerical models of natural landslides to field data on their deposits: *μ*(*U*) = (*μ* − *μ*_w_)/(1 + |*U*|/*U*_w_) + *μ*_w_ with *μ* = 0.84, *μ*_w_ = 0.11, *U*_w_ = 4 ms^−1^ (weakening sliding velocity). This relationship is similar to a friction law derived for weakening by flash heating (Rice 2006; Goren and Aharonov 2007) and observed in laboratory experiments (Di Toro et al. 2011; Rubino et al. 2017; Kuwano 2017).

The real contact between two rough solid surfaces generally occurs over a small fraction of their nominal contact area, on highly stressed micro-contacts. Slip produces frictional heating at the micro-contact scale. If slip is fast enough to prevent heat dissipation by conduction, the micro-contact experiences a significant transient temperature rise that activates thermal effects such as melting, dehydration and other phase transformations (Liu et al. 2015; Lucas et al. 2014). The resulting friction coefficient varies from high values, up to *μ* = 0.7 − 0.8, at low velocities (small volumes) to very small values, down to *μ* < 0.1, for rapid flows (large volumes). Recent experiments with direct microscopic observation of frictional interfaces conducted at sliding velocities from millimetres per second to metres per second show dramatic weakening in the friction coefficient for a wide variety of rock types due to mechano-chemical effects by frictional heating (Kuwano 2017).

**Fig. 16:**
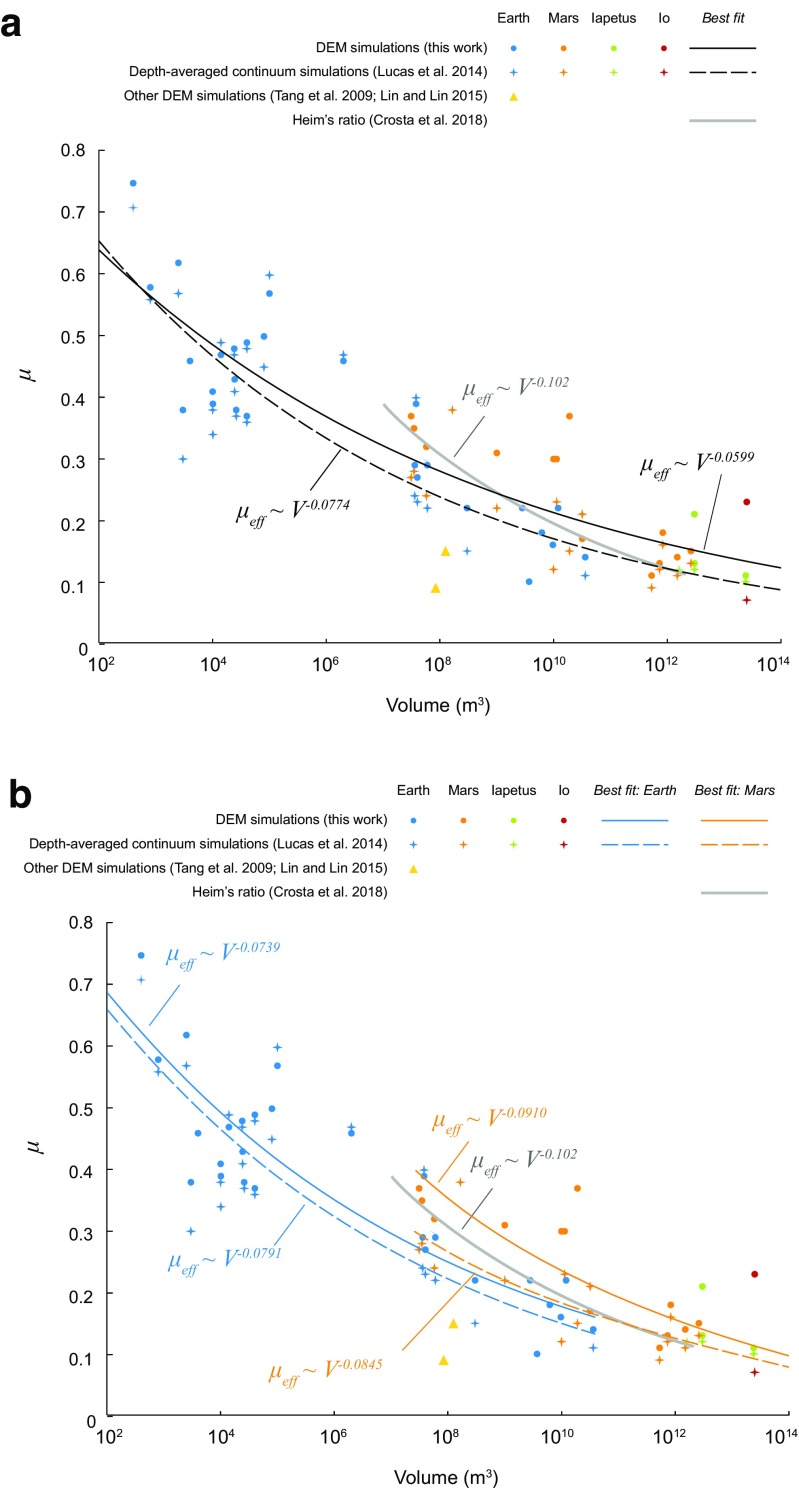
**a** Microscopic (*μ*_m_ = *μ*_c_ = *μ*_w_) friction coefficient as a function of the volume of landslides, as in Fig. 15, compared with the best power-law fit obtained by Crosta et al. (2018) from Heim’s ratios calculated for 222 Martian landslides. The slope of the fit line is steeper than the slope of the fit line obtained in DEM simulations and in depth-averaged continuum models. **b** When the results of simulations and continuum models of terrestrial and Martian landslides are fit separately, the slope of the fit line for the Martian landslides gets closer to the slope of the fit line obtained by Crosta et al. (2018), but the coefficient of determination does not improve (Table 2)

The friction versus volume empirical fit obtained in the DEM simulations and in continuum models is quite close to the best-fit Heim’s ratio calculated by Crosta et al. (2018) from a large Martian landslide dataset (Fig. 16). The slope of the fit line obtained by Crosta et al. (2018) for Mars is, however, steeper than that obtained from our simulations as well as the depth-averaged continuum models, which additionally include landslides from Earth and other bodies (Fig. 16a). In the DEM and continuum models, isolating the Martian landslides from others (Fig. 16b) allows to reproduce better the data fit line of Crosta et al. (2018), suggesting that the frictional behaviour of landslides might be different on different planets. However, as shown in Table 2, when Earth and Mars are considered separately, for continuum models, the coefficient of determination *R*^2^ significantly deteriorates (from 0.85 to 0.69), suggesting that the difference between Earth and Mars may be an artefact due to the small dataset size. In DEM simulations, considering Earth and Mars separately increases *R*^2^ for Earth (0.8 instead of 0.75 when all the solar system data are fit), but decreases it for Mars (0.71 instead of 0.75). These results are difficult to interpret; whether separating Earth data from Mars data improves the fit is hard to tell. Larger datasets and many more simulations are needed for the statistics to be robust and to appreciate whether or not different conditions on different planetary bodies do affect the frictional behaviour of landslides in relation with their volume. The potential causes for such a different behaviour would need to be explored.

**Table 2:** Exponent and coefficient of determination of power-law fits of landslide datasets presented on Fig 16

Power-law fit, volume vs. friction	Solar system		Earth		Mars	
	Exponent	*R* ^2^	Exponent	*R* ^2^	Exponent	*R* ^2^
Simulations	− 0.0599	0.75	− 0.0739	0.8	− 0.091	0.71
Continuum models	− 0.0774	0.85	− 0.0791	0.69	− 0.0845	0.69
Heim’s ratio					− 0.102	

## Conclusion

1. We have numerically investigated the collapse and the spreading of rectangular and axisymmetric columns of grains onto horizontal and sloping planes using the discrete element method. The results quantitatively match the experimental results published so far. They correctly reproduce these experiments during dynamic spreading up to the arrest phase.
2. Quantitative relationships between the column aspect ratio, initial volume and normalised runout distance also agree with laboratory experiments. For the horizontal geometry and slope angles up to 16°, the influence of the volume of the granular mass on the runout distance is negligible. On the contrary, when the slope angle increases (at slopes 19°, 22° and 23° investigated here), the normalised runout does not depend on the aspect ratio only but also clearly depends on the volume involved. This volume dependency is reproduced by the DEM simulations without having to change the microscopic friction coefficient.
3. The discrete element simulations show that the empirical friction coefficients necessary to reproduce the large distances travelled by natural landslides are smaller when the landslide volume (or velocity) increases as it was empirically observed using depth-averaged continuum models. This suggests that frictional weakening for large (i.e. rapid) landslides is necessary whatever the model used and that this phenomenon should reflect a real physical process.
4. No clear difference is observed between the friction weakening of landslides on Earth and on Mars, suggesting that, at leading order, common mechanisms could explain the decrease in friction with volume (or velocity). Whatever the mechanism involved, a friction weakening with volume or velocity makes it possible to reproduce empirically the first-order runout of all landslides.
5. The empirical friction weakening law obtained from fitting DEM simulation with observed runout distances is compatible with flash-heating laws.

Despite the limitation of the simple DEM and depth-averaged shallow models, the quantitative change of the effective friction may help to further identify, using more advanced physical models, the physical origin of this high mobility of large landslides on Earth and on Mars.

## Electronic supplementary material

Supplementary Figure 1 Determination of slope classes used in Fig. 14. (DOCX 112 kb)

Supplementary Table 1 3D discrete element simulation of dam-break rectangular collapses over an inclined bed: parameters used for the simulation of real landslides (Fig. 13). (DOCX 21 kb)

Supplementary Table 2 Source of error bars for Fig. 14. (DOC 53 kb)
