# Supplementary material for: Empirical investigation of friction weakening of terrestrial and Martian landslides using discrete element models
Source: Landslides. 2019 Mar 1;16(6):1121–40. doi: 10.1007/s10346-019-01140-8 (PMC6529039; doi:10.1007/s10346-019-01140-8)
Supplement: Supplementary file 1 — Determination of slope classes used in Fig. 14. (DOCX 112 kb) [file 10346_2019_1140_MOESM1_ESM.docx]

Supplementary Figure 1

Determination of slope classes used in Figure 14


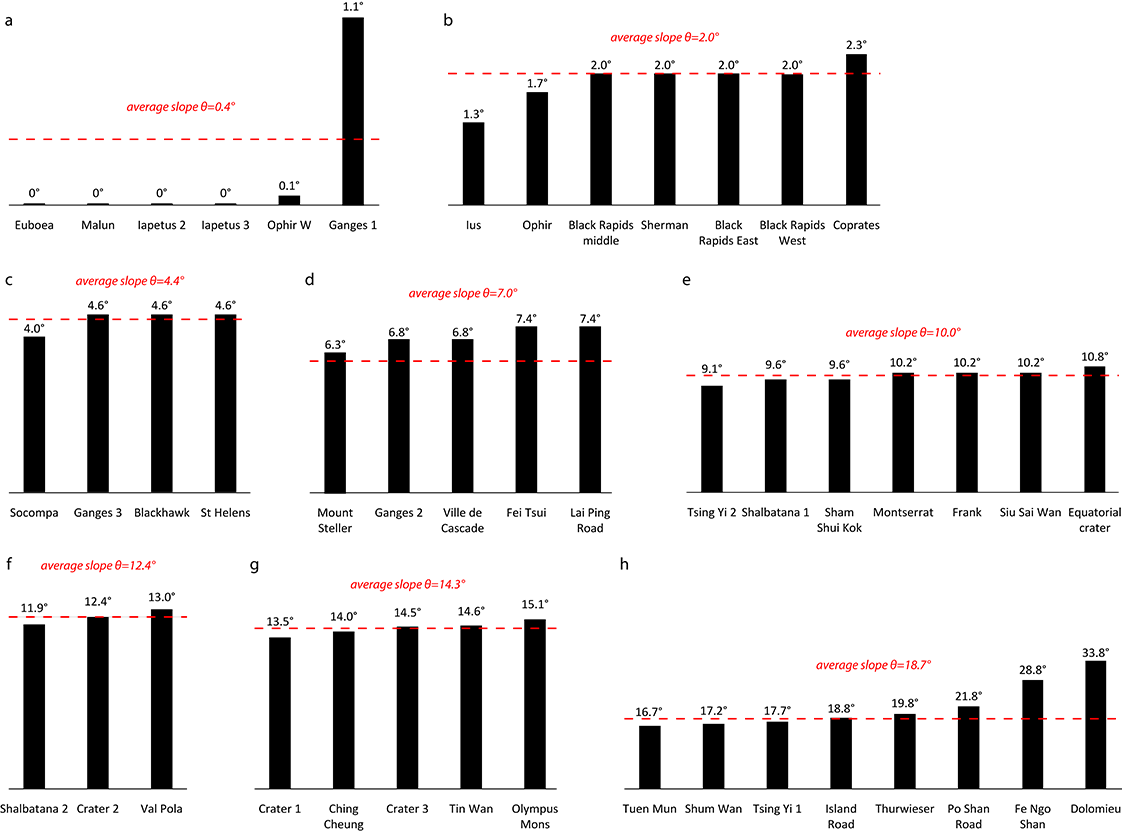


The slope of each landslide is different from the others. In order to compare the propagation of landslides having similar slopes, slope classes need to be defined. The slope angles were grouped into classes in such a way that the deviation between landslide slope and the slope affected to the class is minimal, while keeping a reasonable number of groups. The mean deviation between landslide slope angle and the class slope angle is 0.50°. In the analyzed dataset, but this deviation is significantly larger (3.1°) in the class slope 18.7°.
