# Supplementary material for: Empirical investigation of friction weakening of terrestrial and Martian landslides using discrete element models
Source: Landslides. 2019 Mar 1;16(6):1121–40. doi: 10.1007/s10346-019-01140-8 (PMC6529039; doi:10.1007/s10346-019-01140-8)
Supplement: Supplementary file 2 — 3D discrete element simulation of dam-break rectangular collapses over an inclined bed: parameters used for the simulation of real landslides (Fig. 13). (DOCX 21 kb) [file 10346_2019_1140_MOESM2_ESM.docx]

Supplementary Table 1

3D discrete element simulation of dam-break rectangular collapses over an inclined bed:

parameters used for the simulation of real landslides (Figure 14). Initial slope genometry parameters are from Supplementary Table 2.

| Landslide name | Aspect ratio  *a* | Normalized observed runout | Initial runout  *R_0_* (m) | Initial  height/width  *H_0_* = *W_0_* (m) | Microscopic friction obtained in DEM simulation  *µ_m=_ µ_c=_ µ_w_* | Mean slope  *θ* (°) | Volume of real landslide  (m^3^) |
| --- | --- | --- | --- | --- | --- | --- | --- |
| Island Road | 0.08 | 0.38 | 50 | 4 | 0.58 | 18.8 | 8.00E+02 |
| Ching Cheung | 0.06 | 1.20 | 41.7 | 2.5 | 0.38 | 14 | 3.00E+03 |
| Tsing Yi 2 | 0.24 | 0.81 | 29.2 | 7 | 0.46 | 9.1 | 4.00E+03 |
| Ville de Cascade | 0.22 | 0.85 | 54.5 | 12 | 0.41 | 6.8 | 1.00E+04 |
| Siu Sai Wan | 0.15 | 0.93 | 40 | 6 | 0.39 | 10.2 | 1.01E+04 |
| Fei Tsui | 0.32 | 0.64 | 46.9 | 15 | 0.47 | 7.4 | 1.40E+04 |
| Tsing Yi 1 | 0.13 | 0.86 | 76.9 | 10 | 0.48 | 17.7 | 2.40E+04 |
| Fei Ngo Shan | 0.08 | 2.92 | 40 | 3.2 | 0.62 | 28.8 | 2.50E+03 |
| Tin Wan | 0.15 | 0.99 | 73.3 | 11 | 0.43 | 14.6 | 2.44E+04 |
| Shum Wan | 0.15 | 2.10 | 66.7 | 10 | 0.38 | 17.2 | 2.60E+04 |
| Po Shan Road | 0.13 | 1.60 | 92.3 | 12 | 0.49 | 21.8 | 4.00E+04 |
| Sham Shui Kok | 0.17 | 0.89 | 176.9 | 23 | 0.37 | 9.6 | 4.00E+04 |
| Tuen Mun | 0.13 | 0.87 | 92.3 | 12 | 0.50 | 16.7 | 8.00E+04 |
| Lai Ping Road | 0.18 | 0.38 | 111.1 | 20 | 0.57 | 7.4 | 1.00E+05 |
| Thurwieser | 0.07 | 0.50 | 5000 | 350 | 0.46 | 19.8 | 2.00E+06 |
| Impact crater 1 | 0.07 | 1.85 | 642.9 | 45 | 0.37 | 13.5 | 3.11E+07 |
| Impact crater 2 | 0.08 | 3.39 | 500 | 40 | 0.32 | 12.4 | 5.76E+07 |
| Impact crater 3 | 0.05 | 1.82 | 700 | 35 | 0.35 | 14.5 | 3.49E+07 |
| Frank slide | 0.21 | 2.26 | 1666.7 | 350 | 0.29 | 10.2 | 3.60E+07 |
| Val Pola | 0.08 | 0.32 | 6250 | 500 | 0.39 | 13.0 | 3.80E+07 |
| Montserrat | 0.26 | 6.49 | 692.3 | 180 | 0.27 | 10.2 | 4.00E+07 |
| Mount Steller | 0.08 | 4.10 | 1687.5 | 135 | 0.29 | 6.3 | 6.00E+07 |
| Blackhawk | 0.08 | 0.80 | 10000 | 800 | 0.22 | 4.6 | 0.30E+09 |
| Shalbatana Vallis 2 | 0.05 | 2.55 | 1600 | 80 | 0.31 | 11.9 | 1.00E+09 |
| St Helens | 0.10 | 2.10 | 9000 | 900 | 0.22 | 4.6 | 2.80E+09 |
| Sherman | 0.19 | 6.25 | 5684.2 | 1080 | 0.10 | 2.0 | 3.70E+09 |
| Black Rapids West | 0.21 | 1.45 | 3476.2 | 730 | 0.18 | 2.0 | 6.20E+09 |
| Black Rapids Middle | 0.14 | 1.27 | 5714.3 | 800 | 0.16 | 2.0 | 9.70E+09 |
| Ganges Chasma 3 | 0.44 | 4.55 | 1704.5 | 750 | 0.30 | 4.6 | 9.95E+09 |
| Equatorial Crater | 0.08 | 1.79 | 2500 | 200 | 0.30 | 10.8 | 1.13E+10 |
| Black Rapids East | 0.24 | 1.58 | 4083.3 | 980 | 0.22 | 2.0 | 1.20E+10 |
| Dolomieu Crater flow | 0.20 | 4.07 | 100 | 20 | 0.75 | 33.8 | 4.00E+02 |
| Ganges Chasma 2 | 0.42 | 3.32 | 1190.5 | 500 | 0.37 | 6.8 | 1.90E+10 |
| Shalbatana Vallis 1 | 0.26 | 2.79 | 1538.5 | 400 | 0.17 | 0 | 3.19E+10 |
| Socompa | 0.25 | 5.04 | 8000 | 2000 | 0.14 | 4.0 | 3.60E+10 |
| Ganges Chasma 1 | 0.50 | 4.43 | 6580 | 3290 | 0.11 | 1.1 | 5.30E+11 |
| Coprates Chasma | 0.41 | 7.45 | 10731.7 | 4400 | 0.13 | 2.3 | 7.30E+11 |
| Ophir Chasmа | 0.56 | 3.75 | 10000 | 5600 | 0.18 | 1.7 | 8.33E+11 |
| Ophir Chasma West | 0.53 | 4.58 | 8301.9 | 4400 | 0.14 | 0.1 | 1.50E+12 |
| Ius Chasma | 0.45 | 3.34 | 12888.9 | 5800 | 0.15 | 1.3 | 2.60E+12 |
| Iapetus 2 | 1.60 | 24.6 | 3125 | 5000 | 0.13 | 0 | 3.00E+12 |
| Iapetus 3 | 0.29 | 2.48 | 17241.4 | 5000 | 0.21 | 0 | 3.00E+12 |
| Malun | 0.72 | 6.80 | 8333.3 | 6000 | 0.11 | 0 | 2.40E+13 |
| Euboea Montes | 0.085 | 1.15 | 70588.2 | 6000 | 0.23 | 0 | 2.50E+13 |
