# Supplementary material for: Empirical investigation of friction weakening of terrestrial and Martian landslides using discrete element models
Source: Landslides. 2019 Mar 1;16(6):1121–40. doi: 10.1007/s10346-019-01140-8 (PMC6529039; doi:10.1007/s10346-019-01140-8)
Supplement: Supplementary file 3 — Source of error bars for Fig. 14. (DOC 53 kb) [file 10346_2019_1140_MOESM3_ESM.doc]

**Supplementary Table 2**

Source of field landslide data, including error bars reported on Figure 14.

| **Name** | **Reference** |
| --- | --- |
| Fei Ngo Shan | King (1999) |
| Shalbatana Vallis 2 | Lucas et al. (2014) |
| Impact crater 3 | Lucas et al. (2014) |
| Dolomieu Crater flow | Hibert et al. (2011) |
| Ching Cheung | King (1999) |
| Impact crater 1 | Lucas et al. (2014) |
| Thurwieser | Sosio et al. (2008) |
| Equatorial Crater | Lucas et al. (2014) |
| Impact crater 2 | Lucas et al. (2014) |
| Ganges Chasma 2 | this work |
| Val Pola | Crosta et al. (2007) |
| Blackhawk | Shreve (1987) |
| Island Road | King (1999) |
| Mount Steller | Moretti et al. (2012) |
| Euboea Montes | Lucas et al. (2014) |
| St Helens | Voight et al. (1983) |
| Ganges Chasma 3 | This work |
| Shalbatana Vallis 1 | This work |
| Tsing Yi 1 | King (1999) |
| Tuen Mun | King (1999) |
| Po Shan Road | King (1999) |
| BRG-middle | Shugar et al. (2011) |
| Sui Sai Wan | King (1999) |
| Shum Wan | King (1999) |
| Tin Wan | King (1999) |
| Sham Shui Kok | King (1999) |
| Lai Ping Road | King (1999) |
| Sherman | McSaveney (1978) |
| BRG-west | Shugar et al. (2011) |
| Frank slide | *R_n_*: Hungr (1981), Pedrazzini et al. (2011); *a*: this work |
| Ville de Cascade | King (1999) |
| Tsing Yi 2 | King (1999) |
| BRG-east | Shugar et al. (2011) |
| Socompa | *R_n_*: Kelfoun & Druitt (2005); *a*: this work |
| Montserrat | Young et al. (1998) |
| Iapetus 3 | Singer et al. (2012) |
| Fei Tsui | King (1999) |
| Coprates Chasma | This work |
| Ius Chasma | Lucas et al. (2011) |
| Ganges Chasma 1 | this work |
| Ophir Chasma West | Lucas et al. (2011) |
| Ophir Chasmа | Lucas et al. (2011) |
| Malun | Lucas et al. (2014) |
| Iapetus 2 | Singer et al. (2012) |

The error on aspect ratio *a* estimated in this work is from uncertainty on initial "dam" radius *R_0_* (defined in Figure 4) from interpretation of image data (MRO/CTX) and topography data (Mars Express/HRSC DEM mosaic (Gwinner et al., 2009, Photogramm Eng Remote Sensing 75(9):1127–1142) or, when not available, MGS/MOLA DEM). The error on runout distance *R_n_* is estimated from MRO/CTX images.

**Cited references:**

Crosta GB, Frattini P and Fusi N (2007) Fragmentation in the Val Pola rock avalanche, Italian Alps. J Geophys Res. 112, F01006

Hibert C, Mangeney A, Grandjean G and Shapiro N (2011) Slopes instabilities in the Dolomieu crater, la Réunion island : from the seismic signal to the rockfalls characteristics. J Geophys Res 116:F04032

Hungr O (1981) Mobility of rock avalanches. Rept Nat Res Inst Earth Sci Disaster Prevention, Tsukuba, Japan, 46:11-20

Kelfoun K and Druitt TH (2005) Numerical modeling of the emplacement of Socompa rock avalanche, Chile. J. Geophys Res 110, B12202

King J P (1999) Natural terrain landslide study, The natural terrain landslide inventory. GEO Report No. 74, Hong Kong, Geotechnical Engineering Office, 127 p.

Lucas A, Mangeney A, Mège D and Bouchut F (2011) Influence of scar geometry on landslide dynamics and deposits: application to Martian landslides. J Geophys Res 116:E10001, doi:10.1029/2011JE003803

Lucas A, Mangeney A and Ampuero JP (2014) Frictional weakening in landslides on Earth and on other planetary bodies. Nature Comunications 5:3417, doi:10.1038/ncomms4417

McSaveney MJ (1978) Sherman Glacier rock avalanche, Alaska, U.S.A. In: Voight B (Ed.) Rockslides and avalanches, 1. Natural Phenomena. Elsevier, Amsterdam, 197-258

Moretti L, Mangeney A, Capdeville Y, Stutzmann E, Huggel C, Schneider D, Bouchut F (2012) Numerical modeling of the Mount Steller landslide flow history and of the generated long period seismic waves. Geophys Res Lett 39:L16402. doi:10.1029/2012GL052511

Pedrazzini A, Jaboyedoff M, Troese CR, Langenberg CW and Moreno F (2011) Structural analysis of Turtle Mountain: origin and influence of fractures ni the development of rock failures. In, Jaboyedoff M (ed) Slope Tectonics: London, Geol Soc Sp Publ 351, 163–183. doi:10.1144/SP351.9

Singer K, McKinnon W, Schenk P, and Moore J (2012) Massive ice avalanches on Iapetus caused by friction reduction during flash heating. Nature Geoscience, 5(8):574-578

Shugar DH and Clague JJ (2011) The sedimentology and geomorphology of rock avalanche deposits on glaciers. Sedimentology 58:1762–1783. doi: 10.1111/j.1365-3091.2011.01238.x

Shreve RL (1987) Blackhawk landslide, southwestern San Bernardino County, California Hill. In: Hill, ML (ed) Cordilleran Section of the Geological Society of America, Centennial Field Guide: Boulder, Colorado, Geological Society of America 1:109-114

Sosio R, Crosta GB and Hungr O (2008) Complete dynamic modeling calibration for the Thurwieser rock avalanche (Italian Central Alps). Eng Geology 100(1-2):11-26

Voight B, Janda R, Glicken H and Douglass P (1983) Nature and mechanics of the mount St. Helens rockslide-avalanche of May 1980. Géotechnique 33:243-273

Young SR, Sparks RSJ, Aspinall WP, Lynch LL, Miller AD, Robertson REA and Shepherd JB (1998) Overview of the eruption of soufriere hills volcano, Montserrat, 18 July 1995 to December 1997. Geophys Res Lett 25(18):3389-3392
